# Quantitative proteomics signature profiling based on network contextualization

**DOI:** 10.1186/s13062-015-0098-x

**Published:** 2015-12-15

**Authors:** Wilson Wen Bin Goh, Tiannan Guo, Ruedi Aebersold, Limsoon Wong

**Affiliations:** School of Pharmaceutical Science and Technology, Tianjin University, 92 Weijin Road, Tianjin City, 300072 China; Center for Interdisciplinary Cardiovascular Sciences, Harvard Medical School, Boston, USA; Department of Biology, Institute of Molecular Systems Biology, ETH Zurich, Zurich, Switzerland; Faculty of Science, University of Zurich, Zurich, Switzerland; School of Computing, National University of Singapore, Singapore, Singapore

**Keywords:** Proteomics, Networks, Quantitative Proteomics Signature Profiling (qPSP), Bioinformatics, SWATH, Systems Biology

## Abstract

**Background:**

We present a network-based method, namely quantitative proteomic signature profiling (qPSP) that improves the biological content of proteomic data by converting protein expressions into hit-rates in protein complexes.

**Results:**

We demonstrate, using two clinical proteomics datasets, that qPSP produces robust discrimination between phenotype classes (e.g. normal vs. disease) and uncovers phenotype-relevant protein complexes. Regardless of acquisition paradigm, comparisons of qPSP against conventional methods (e.g. t-test or hypergeometric test) demonstrate that it produces more stable and consistent predictions, even at small sample size. We show that qPSP is theoretically robust to noise, and that this robustness to noise is also observable in practice. Comparative analysis of hit-rates and protein expressions in significant complexes reveals that hit-rates are a useful means of summarizing differential behavior in a complex-specific manner.

**Conclusions:**

Given qPSP’s ability to discriminate phenotype classes even at small sample sizes, high robustness to noise, and better summary statistics, it can be deployed towards analysis of highly heterogeneous clinical proteomics data.

**Reviewers:**

This article was reviewed by Frank Eisenhaber and Sebastian Maurer-Stroh.

**Open peer review:**

Reviewed by Frank Eisenhaber and Sebastian Maurer-Stroh.

**Electronic supplementary material:**

The online version of this article (doi:10.1186/s13062-015-0098-x) contains supplementary material, which is available to authorized users.

## Background

Mass spectrometry (MS)-based proteomics has become a central technology for life sciences research. With extensive fractionation of samples and data-dependent acquisition (DDA) MS or so-called shotgun/discovery proteomics, near-complete proteome coverage is now achievable for a number of prokaryotic and eukaryotic species, including *Schizosaccharomyces pombe* (fission yeast) [1, 2], *Saccharomyces cerevisiae* (budding yeast) [3], *Leprospira interrogans* [4], *Escherichia coli* [5], *Mycobacterium tuberculosis* [6], *Drosophila melanogaster* [7], *Caenorhabditis elegans* [8], *Arabidopsis thaliana* [7–9], human cells [10, 11] and tissues [12, 13].

However, to obtain functional understanding of dynamic biological systems, proteins have to be quantified consistently across different states or samples. In data-dependent acquisition (DDA) MS or shotgun proteomics, peptides eluting from the column are first surveyed and selected for fragmentation based on intensity using a quasi-stochastic heuristic. Different peptides are selected for fragmentation in different runs, leading to stochastic and inconsistent quantitative information. When applied to relatively large sample cohorts, many proteins could not be consistently quantified across samples. This inconsistency was not due to these proteins being absent or below the limit of detection; rather, these proteins were simply not analyzed.

Recent technological advancements in proteomics [14, 15] have led to a surge in data-independent acquisition (DIA) methods such as MSE [16] and SWATH [17], which fragment precursors and record fragment ions independent of their stoichiometry in the sample thereby offering more consistent protein quantification across samples. While DIA methods are broadly similar, they differ in the way the data are analyzed. In particular, SWATH acquires data by repeatedly cycling through precursor isolation windows, referred to as SWATH windows, within a predefined m/z range covering the whole mass range of most MS-measurable precursors [17]. The SWATH strategy thus creates an exhaustive multi-windowed SWATH map for every sample via a single injection. When coupled with an emerging sample-preparation method, Pressure Cycling Technology (PCT), the SWATH workflow could be used to reproducibly digitize the proteomes of small sample quantities in a high-throughput fashion [18]. A unique benefit of SWATH-MS is the potential to align fragment ion signals across multiple samples, producing a data matrix that is nearly complete. This is similar in principle to “match between runs” analysis available in MaxQuant for DDA data matrix [19], but with higher accuracy because the feature alignment in SWATH is based on MS2 peak groups and retention time. Nevertheless, SWATH data are noisier than most DDA data due to concurrent fragmentation and acquisition of a larger number of precursors [17].

Network-based methods can mitigate noise, consistency and coverage issues in proteomics data. Proteome coverage can be expanded, alongside recovery of undetected proteins by identifying proteins that are closely associated with already-identified proteins [20–22]. Consistency and noise issues can be dealt with by approaches such as Proteomics Signature Profiling (PSP) where inconsistent protein identifications between samples are transformed into more consistent features by measuring the hit-rates of the identified proteins against predicted network clusters and/or known complexes [20, 23, 24].

Traditional DDA or shotgun proteomics produces sparse data that is highly inconsistent between samples, even from the same phenotype class. The lack of a complete data matrix means that most statistical methods cannot be effectively used. PSP resolves this problem by transforming the detected proteins into a vector of hit-rates by comparing against protein complexes [20, 23]. Recent advances in proteomics technologies have yielded higher-quality data matrices with more reliable quantitation. In particular, these new methods generate near-complete data matrices. Since PSP uses only the detection/non-detection of proteins, it cannot be used directly on these data matrices. However, recent work in genomics revealed that meaningful class-discriminative signal is enriched amongst the top n% of abundance-ranked genes [25, 26]. Using this principle, we extend PSP to a quantitative variant (qPSP) where protein-abundance information is incorporated. We demonstrate its effectiveness using two datasets, viz. a recently published SWATH MS renal cancer dataset [18] and a large high-quality DDA dataset for colorectal cancer [27].

## Methods

### Proteomics dataset 1 (DDA) --- Colorectal Cancer (CR)

The colorectal cancer (CR) study of Zhang et al. [27] using high-resolution LC-MS/MS is suitable for benchmarking (Additional file 1). This dataset contains 90 colorectal cancer samples derived through TCGA’s Biospecimen Core Resource (BCR), and 30 normal (non-matched) samples obtained from screening colonoscopies. Particular emphasis was placed on ensuring data quality in this study: For every 5 CR samples, benchmark quality controls (QCs) from one basal and one luminal human breast tumor xenograft were analyzed. Analysis of data from benchmark QCs allows checks for platform reproducibility and also evaluation of data normalization methods. For exhaustive spectral coverage, both standard search and spectral-library matching methods were used. For standard search, the raw spectra were compared against patterns of in silico fragmented peptides from the RefSeq human protein sequence database (V.54) [28] using Myrimatch v2.1.87 [29]. Spectral-library matching was performed using Pepitome [30] and referenced against the NIST database where the m/z and rt profiles of actual peptides were captured and stored [31]. Peptide-identification stringency was set at FDR of 2 % for higher sensitivity. However, for protein assembly, a minimum of 2 unique peptides per protein is essential for a positive identification (3899 proteins with a protein-level FDR of 0.43 %). To limit the number of data holes, only proteins supported by 95 % of samples were kept in the final matrix (3609 proteins).

Proteins are quantified via spectral count, which is the total number of MS/MS spectra acquired for peptides from a given protein. In this study, we excluded label-based quantitation such as iTRAQ and TMT [32] due to severe sample-size constraints. Although it is possible to up-scale TMT up to 10 or 18 samples, this is still relatively small and unsuitable for benchmarking of new methods [32].

### Proteomics dataset 2 (DIA) --- Renal Cancer (RC)

DIA methods are relatively new in the field, and large applicable datasets are not easy to obtain. As an instance of DIA proteomics, we used the SWATH datasets from Guo *et al.* in their study of clear cell renal carcinoma or renal cancer [33]. The first one (RC-C) is composed of 12 SWATH runs from a human kidney test tissue digested in quadruplicates and each digest analyzed in triplicates using a tripleTOF 5600 mass spectrometer (AB Sciex) (Additional file 2). The second data set (RC) contains 24 SWATH runs from 6 pairs of non-tumorous and tumorous clear-cell renal carcinoma (ccRCC) or more simply, RC tissues, which have been swathed in triplicates (Additional file 3).

Although technical duplicates were present in each phenotype class, we opted to analyze them together: If technical duplicates gave rise to high random variation, then the technical replicates would not group together; this would indicate that the data-acquisition platform was unstable. Moreover, if technical variability is present, it would impede the ability of less-robust algorithms to detect biological signal. Since we are interested to know the performance of the qPSP in the presence of noise (from biological and technical variation) in the data, it should not matter so much that the technical replicates were grouped together in the study.

All SWATH maps were analyzed using OpenSWATH [34] against a spectral library containing 49959 reference spectra for 41542 proteotypic peptides from 4624 reviewed SwissProt proteins [33]. The library was compiled using DDA data of the kidney tissues in the same mass spectrometer. Protein isoforms and protein groups were excluded from this analysis.

For RC-C, 2331 proteins were quantified across the 12 SWATH maps of the first data set with a peptide and protein false-discovery rate (FDR) lower than 1 %. In the original RC, 2375 proteins were quantified across all samples with a precursor FDR below 0.1 % and 32 % sparsity. The high level of sparseness was likely due to higher noise and poorer alignments between features. To improve consistency in the data, we relaxed the retention time (RT) alignment criteria using TRansition of Identification Confidence (TRIC) (version r238); specifically, we allowed a wider maximal RT difference (max_RT = 30) but increased the precursor FDR to 0.001 to limit false matches. The two most intense peptides were used to quantify proteins. Finally, protein FDR was set to 0.01 with 3123 proteins reported.

### Alpha-stability analysis

We define “alpha” as the value used to subset a ranked protein list based on abundance. The alpha stability is a measure of alpha-protein rank consistency between samples in the same phenotype class (e.g. normal samples from the normal class) ---i.e., whether the alpha proteins are similar across same-class members. Evaluation of the alpha stability (i.e. how consistent protein rankings are) provides insight into inter-sample variability as well as noise levels.

We used Alpha-Stability Analysis (ASA) to evaluate protein rank stability across samples to obtain insights into technical and biological variability. ASA first sorts the proteins in each sample by protein abundance so that the most highly expressed proteins are ranked first in that sample. The top n% proteins in the sorted list of each sample constitute the set of alpha proteins of that sample. ASA then cross-compares the sets of alpha proteins between all samples using the Jaccard distance (i.e. the intersection of two sets of proteins over the union of proteins in the same two sets).

### Protein-complex analysis

Although subnets or clusters can be predicted from large biological networks, it is found that real biological complexes are biologically more coherent [22]. Thus, we used protein complexes from the CORUM database which contains manually annotated protein complexes from mammalian organisms. [35] Some complexes in CORUM are very small and could give rise to high hit-rate fluctuations. Thus we considered 600 protein complexes of at least size 5.

### Implementation of qPSP (Quantitative Proteomics Signature Profiling)

**Fig. 1:**
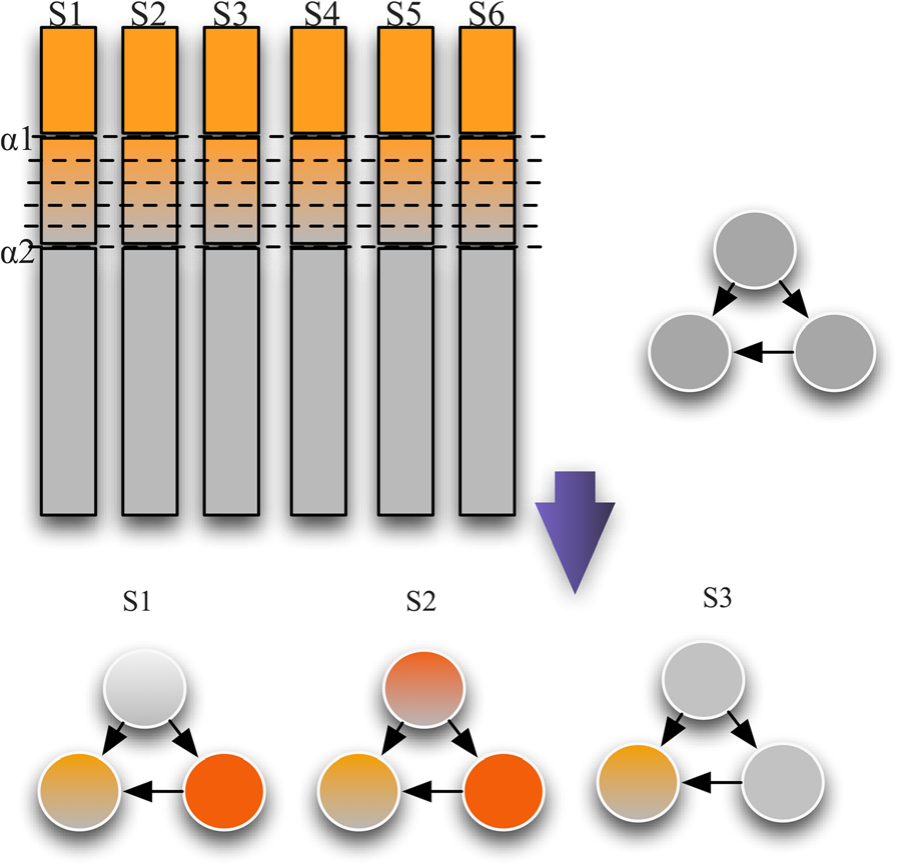
Schematic demonstrating qPSP’s fuzzification procedure. First, alpha1 at top 10 % was defined. An alpha2 was defined from top 10-20 %. To place less confidence in the lower-scoring alpha2, proteins that fall within this range were grouped into 5 bins with descending weights. The modulated hit-rates for each sample could then be used for generating each sample’s proteomic signature profile

We assume that proteins with higher abundance are measured with higher accuracy in the mass spectrometer. qPSP first sorts proteins in each sample based on their measured abundance, and selects the most abundant proteins above a certain percentile (the value is denoted as alpha1). A second percentile value (defined as alpha2) is set to extend the list of proteins for improving sensitivity while maintaining precision [25]. To penalize lower-ranked proteins, proteins are assigned different weights based on which percentiles they fall under (Fig. 1).

By default, we set alpha1 as top 10 %, and alpha2 as top 10-20 %. Rank-based weighting was achieved via discretization of the continuous range from top 10–20 % into four bins: 10–12.5 % (weight 0.8), 12.5 % to 15 % (0.6), 15–17.5 % (0.4), 17.5 to 20 % (0.2). All other proteins outside of alpha2 (viz. remaining proteins) have a weight of 0 (and are thus ignored). Alpha1 proteins are assigned the full weight of 1. For convenience, proteins with non-zero weight are referred to as alpha proteins. For each sample, a vector of hit-rates is produced by considering the overlaps of the alpha proteins against a vector of complexes. To see how this works, let’s say we have a sample from class A (S_A_) and a vector of complexes of length n. For each complex C_i_ in the complex vector, the hit-rate is the intersection of proteins in S_A_ and C_i_ , modulated by the proteins’ weights (i.e. an alpha1 protein counts as 1 in the intersection, an alpha2 protein of weight 0.8 counts as 0.8 in the intersection, and so on), over the total number of proteins in C_i_. We let the hit-rate for S_A_ in Ci be H(S_A,_ C_i_). Therefore, the vector of hit-rates for sample S_A_ is H_SA_ = <H(S_A,_ C_1_)…, H(S_A,_ C_n_)>. This vector of hit-rates is the sample’s subnet signature profile.

To perform feature selection (where a feature is a protein complex in this instance), for each complex C in the complex vector, we compare two lists against each other, HA = <H(A_1_,C),…, H(A_m_,C) > and HB = <H(B_1_,C),…, H(B_n_,C)>, where A and B are phenotype classes of sizes m and n respectively. The t-statistic between HA and HB is computed by the standard formula:

$$ t\_ score=\frac{\overline{HA}-\overline{HB}}{S_{HA,HB}\sqrt{\frac{1}{n}+\frac{1}{m}}} $$

where

$$ {S}_{HA,HB}=\sqrt{\frac{\left(m-1\right){S_{HA}}^2-\left(n-1\right){S_{HB}}^2}{m+n-2}} $$

If the t-statistic is significant (i.e. its associated p-value is lower than 0.05), then C is differentially expressed between classes A and B.

There are several adjustable parameters that determine the endpoint measure, which we examined in the following, including the values of alpha1 and alpha2, and the directionality of alpha values (i.e. top n% or bottom n%).

### Consistency Benchmarking

As a match against a commonly used form of network-enrichment approach, we compared qPSP against the standard hypergeometric enrichment method (hyp_geo). The hyp_geo comprises 2 parts. In the first part, differential proteins are first selected using the unpaired two-sided t-test (*P* ≤0.05). To do this, a t-statistic (*T*_*p*_) is calculated for each protein *p* by comparing the z-normalized expression scores between phenotype classes *C1* and *C2*, with the assumption of unequal variance between the two classes [36], as given below:

$$ {T}_p=\frac{{\overline{x}}_1-{\overline{x}}_2}{\sqrt{\frac{s_1^2}{n_1}+\frac{s_2^2}{n_2}}} $$

where ${\overline{x}}_j$ is the mean abundance level of the protein *p*, *s*_*j*_ is the standard deviation and *n*_*j*_ is the sample size, in class *Cj*. The *T*_*p*_ is compared against the nominal t-distribution to calculate the corresponding *p*-value. A feature is deemed significant if *p*-value ≤ threshold (where threshold is set to 0.05).

In the second part of hyp_geo, the significant proteins from the first part are compared against CORUM complexes using the hypergeometric test. Given a total number of proteins *N*, with *B* of these belonging to a complex and *n* of these proteins in the test set (i.e. differential), the probability *P* that *b* or more proteins from the test set are associated by chance with the complex is given by:

$$ P\left(X\ge b\right)={\displaystyle \sum_{i=b}^{\min \kern0.5em \left(n,B\right)}\frac{\left(\begin{array}{l}n\\ {}i\end{array}\right)\left(\begin{array}{l}N-n\\ {}B-i\end{array}\right)}{\left(\begin{array}{l}N\\ {}B\end{array}\right)}} $$

The complex is deemed significant if P(*X* ≥ *b*) ≤ threshold (where threshold is set to 0.05).

To analyze reproducibility and stability, for both qPSP and hyp_geo, we took random samplings of sizes 4, 6 and 8 from both normal and cancer classes 1000 times to generate a series of binary matrices, where rows are simulations, a value of 1 indicates a complex is significant, and 0 otherwise. Each binary matrix is used for comparing stability and consistency of significant features predicted by qPSP or hyp_geo.

Three evaluations on the binary matrix can be performed: 1/row-wise comparisons based on the Jaccard coefficient to evaluate cross-simulation similarity, 2/row summation to evaluate the distribution of the number of selected significant complexes, and 3/column summation to evaluate the persistence and stability of each selected significant complex.

### Class-discrimination stability

We can analyze the strength of class discrimination in hierarchical clustering using the bootstrap approach PVCLUST [37]. For each cluster in hierarchical clustering, scores (between 0 and 100) are calculated via multi-scale bootstrap resampling. PVCLUST provides two types of scores: AU (Approximately Unbiased) and BP (Bootstrap Probability). AU is a less biased calculation approach than BP; both are shown in the PVCLUST bootstrap trees. Clusters that are highly stable (95 and above for AU scores) are boxed in red.

## Results and discussions

### qPSP improves signal in proteomics data

We first evaluated the signal enhancement and false-positive rate in qPSP using the RC-C data set that is comprised of technical and biological replicates. Since there is only one true underlying class in RC-C, any reported significant complex by splitting RC-C into two groups is a false positive.

**Fig. 2:**
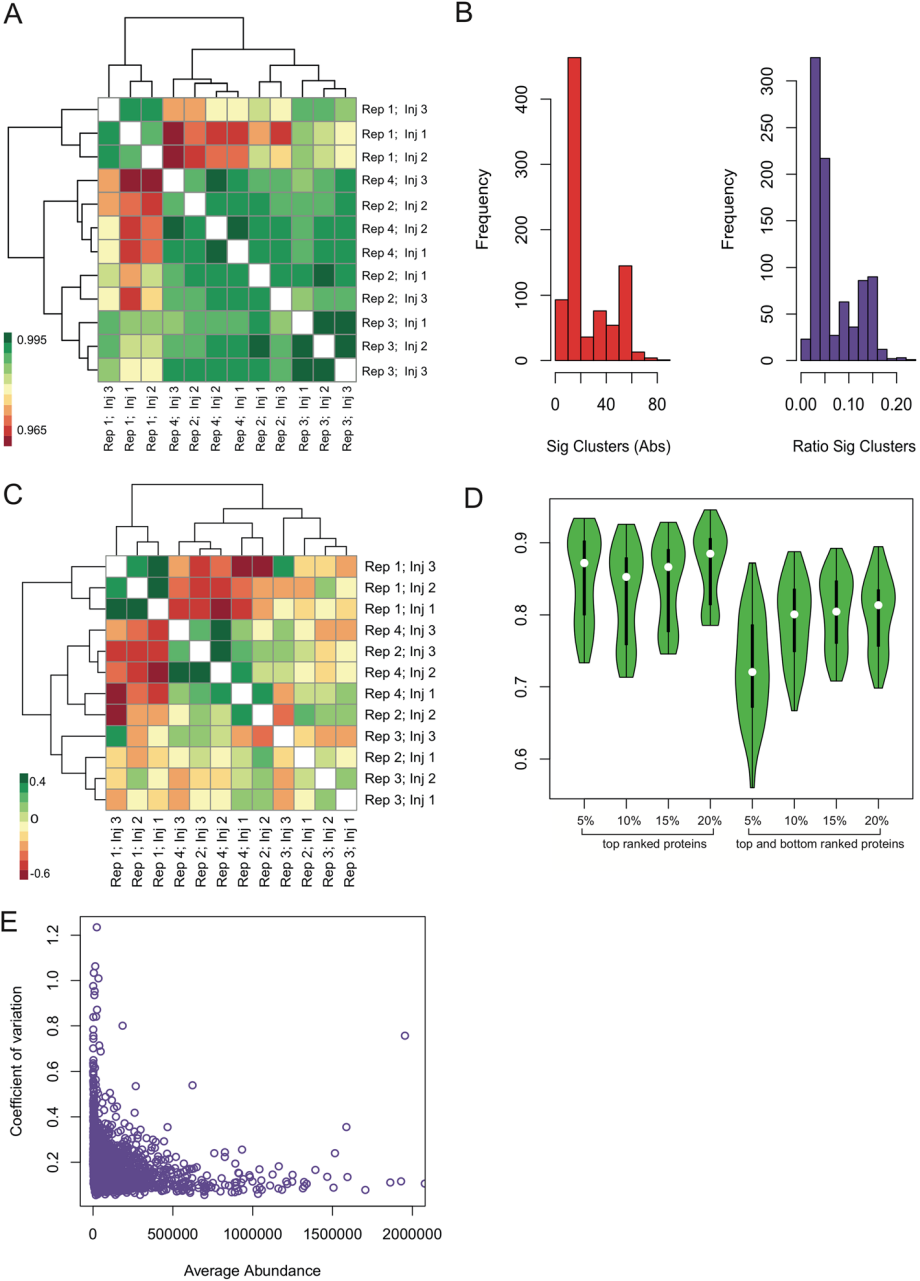
Benchmarks on control data comprising technical and biological replicates. **a** Similarity analysis of controls across all proteins without network contextualization. Green corresponds to similarity while red corresponds to dissimilarity. Samples are clustered using hierarchical clustering using Euclidean distance and Ward’s linkage. **b** Stability analysis of controls at different cut-offs. Y-axis: Jaccard similarity. X-axis: categorical variables for top n% alphas and top n% top and bottom alphas. The top alphas were very stable, ranging between 85 to 88 % agreement rates. On the other hand, the inclusion of the bottom alphas creates increased instability. This led to a significant drop in agreement rates (70–80 %). The lack of instability at the bottom alphas suggested that although under-expressed proteins might be interesting, noise levels are also very high. **c** Coefficient of variation versus average abundance for each protein. Lower-abundance proteins exhibited higher instability. This further supports the results from panel D, that bottom alphas should not be used in qPSP. **d** Similarity maps for biological and technical replicates (based on Pearson correlation of the qPSP’s of these replicates). The controls were largely similar to each other. Rep 1 (Inj 1,2 and 3) was slightly different from the other digestions. But on the whole, similarities ranged between 97 to 99.5 %. **e** False-positive analysis controls. Controls were randomly assigned into two groups of equal size, and qPSP analysis was performed 10000 rounds. For each round, the number of significant clusters (5 % FDR) was determined. The histograms showed that the number of false positive was well within the expectation levels (Sig Clusters Abs E-level: 19, Observed level: 16; Ratio Sig Clusters E-level: 0.05, Observed level: 0.04)

Figure 2a shows the Pearson correlation scores between RC-C samples based on protein abundance levels. Overall, the correlations---these are mostly below 0.4---are lower than expected given that the samples were in fact replicates. This suggests there is considerable biological and technical noise within the SWATH data set.

Next, we applied qPSP to RC-C using various thresholds of alpha (top 5, 10, 15 and 20 %) (Additional file 4: Figures S1A and B). As shown in Fig. 2b, the Jaccard coefficient (the intersection divided over the union) between samples was preserved at different alphas ranging from 5 % to 20 %. Our analyses also showed that inclusion of bottom alphas (i.e., taking the bottom n% instead of from the top) compromised the performance of qPSP significantly, indicating that inclusion of low-abundance proteins led to high instability. This is not surprising since low-abundance proteins have very high coefficients-of-variation (COV) (Fig. 2c). Furthermore, in contrast to hyp_geo, the Pearson correlations between RC-C samples based on hit rate vectors are about 0.99 (Fig. 2d). Thus, qPSP improves signal within replicates.

We also evaluated the false-positive rate of qPSP. RC-C samples were randomly assigned into 2 groups 1000 times, and evaluated by the number of false predictions made. The distribution of false positives across simulations (Fig. 2e) showed that the false-positive rate for qPSP fell well within the expectation values (Observed median value = 16, Expected =19).

### qPSP is theoretically robust to experimental noise

Noise is a confounding factor for most experimental data. We demonstrate here the t-statistic used in qPSP is independent of the false-detection rate, so long as the false-detection rate is the same between the control and test state.

Let the false-detection rate be r, where 0 < r < 1. Let u and v (0 ≤ u, v ≤ 1) respectively be the actual hit rate (*i.e.,* not due to false-positive proteins) expected of a complex C in phenotype-A and phenotype-B patients. Suppose there are k proteins in C. Next, let x and y be the number of false detections in typical phenotype-A and phenotype-B patients. Then r = x/(x + u*k) = y/(y + v *k).

Since the actual hit rate (sans false detections) of C expected in phenotype-A patients is u, the total actual hits in the complex expected in phenotype-A patients is u*k. Since r = x/(x + u*k), we have x = r*u*k/(1-r). Thus, the expected value of the observed hit rate of C in phenotype-A patients is HA = total actual hits + false positives = u*k + r*u*k/(1-r) = u*k/(1-r). Similarly, the expected value of the observed hit rate of C in phenotype-B patients is HB = v*k/(1-r).

The t-score generated by comparing phenotype-A and -B patients is independent of r. To show this, first consider the estimator of the common standard variation S_HA,HB_ between A and B:

$$ \begin{array}{l}{S}_{HA,HB}=\sqrt{\frac{\left(m-1\right){S}_{HA}^2+\left(n-1\right){S}_{HB}^2}{m+n-2}}\\ {}=\sqrt{\frac{\left(m-1\right)\operatorname{var}\left[u*k/\left(1-r\right)\right]+\left(n-1\right)\operatorname{var}\left[v*k/\left(1-r\right)\right]}{m+n-2}}\\ {}=\left(\frac{k}{1-r}\right)\sqrt{\frac{\left(m-1\right)*\operatorname{var}(u)+\left(n-1\right)*\operatorname{var}(v)}{m+n-2}}\\ {}=\left(\frac{k}{1-r}\right){S}_{u,v}\end{array} $$

The t-score is given as:

$$ \begin{array}{l}t\_ score=\frac{\overset{-}{HA}-\overset{-}{HB}}{S_{HA,HB}\sqrt{\frac{1}{n}+\frac{1}{m}}}=\frac{E\left[u*k/\left(1-r\right)\right]-E\left[v*k/\left(1-r\right)\right]}{\left(\frac{k}{1-r}\right){S}_{u,v}\sqrt{\frac{1}{n}+\frac{1}{m}}}\\ {}=\frac{\left(\frac{k}{1-r}\right)\left(E(u)-E(v)\right)}{\left(\frac{k}{1-r}\right){S}_{u,v}\sqrt{\frac{1}{n}+\frac{1}{m}}}=\frac{E(u)-E(v)}{S_{u,v}\sqrt{\frac{1}{n}+\frac{1}{m}}}\end{array} $$

Hence, the *t*-score is independent of the false-detection rate, r. The empirical p-values are based on the distribution of t-scores. Since t-score is independent of r, it follows that the p-value is also independent of it.

Therefore, the *t*-score is independent of the noise levels so long as the false-detection rate is *the same* between the phenotype classes. Under this assumption, qPSP is expected to be robust to noise and thus useful for analyzing data with higher noise levels but greater proteome coverage (lower FDR cut-offs). This can be useful for improving sensitivity while maintaining reasonable signal-to-noise levels.

Of course, the proof above makes also the assumptions that m and n are about the same size, and the standard deviation for each population is more or less the same. While this assumption may not always hold true, using CR data, we find that qPSP is robust to noise in practice (see section “qPSP is immune to noise in SWATH data”). Moreover, an analogous result can be obtained (using essentially the same proof) when t-score with unequal variance is used instead.

### qPSP discriminates control and cancer samples --- RC and CR

**Fig. 3:**
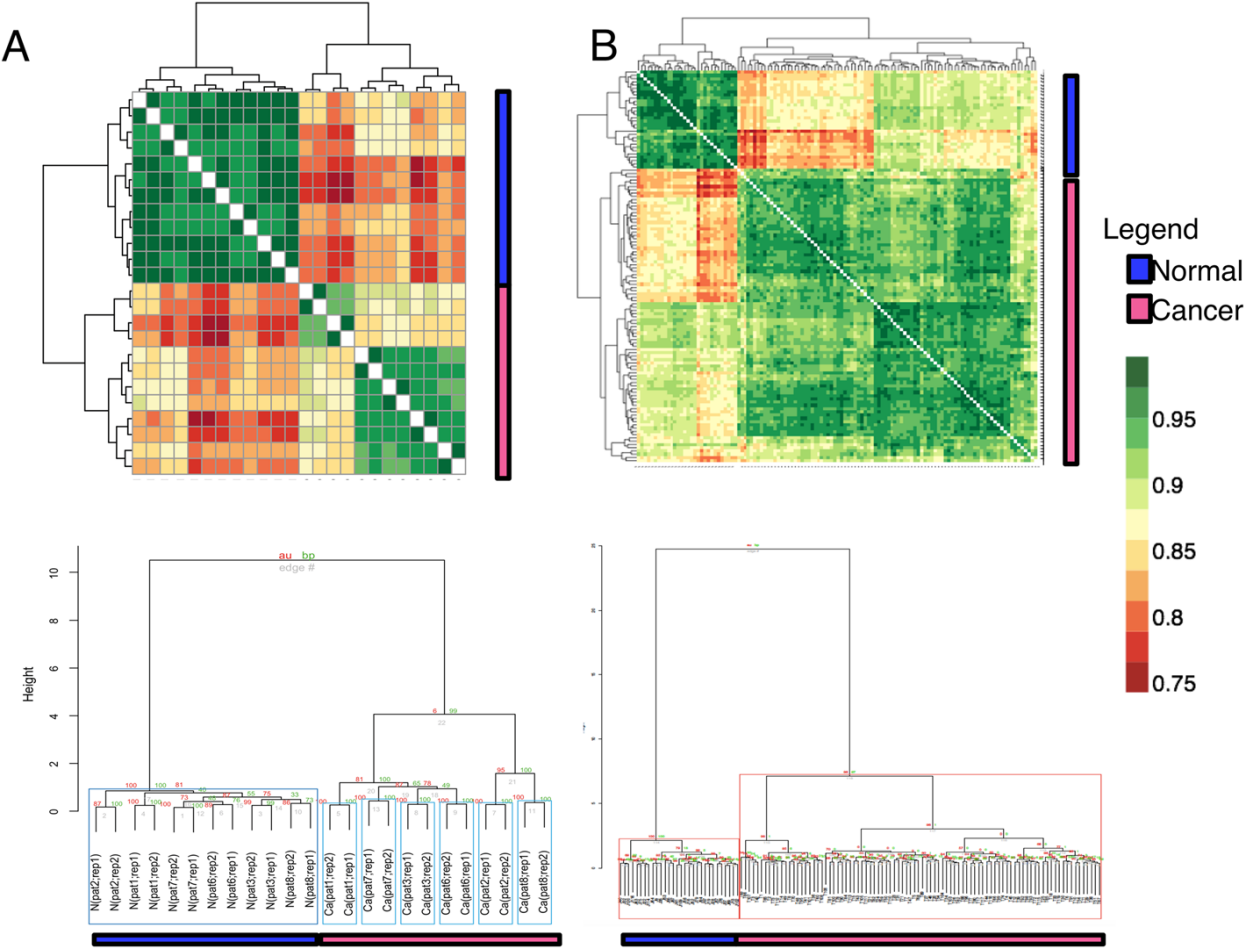
qPSP strongly discriminates sample classes for renal cancer (**a**) and colorectal cancer (**b**). Clustered similarity maps at the top row showed specific and consistent segregation of non-cancer and cancer samples. The trees below the heatmaps are from bootstrap analysis (PVCLUST), which demonstrates that the discrimination between sample classes based on qPSP hit-rates is highly stable

We evaluate whether qPSP allows robust distinction of phenotype classes (normal, cancer) using both the renal cancer (RC) derived from DIA proteomics (Fig. 3a) and the colorectal cancer (CR) derived from DDA proteomics (Fig. 3b). Based on the similarity maps between control and cancer samples using Pearson correlation of hit-rate vectors (Figs. 3a and b; top row), it is evident that cancer samples are well separated from control samples. The correlation score range appeared narrow (between 0.75 and 1.00) since no prior feature selection was explicitly performed between normal and cancer classes. For RC (Fig. 3a), we observed a further sub-grouping in the cancer group. It turned out that the smaller grouping, consisting of patients 2 and 8, belongs to a severe form (Both patients 2 and 8 are deceased).

Using qPSP, the strong discrimination between normal and cancer classes is also clearly demonstrated in the PVCLUST-generated HCL tree (Euclidean distance; Ward’s linkage) where bootstrap resampling confidently separated them into two distinct groups (Fig. 3a and b; bottom row), which are encapsulated in highlighted boxes (AU scores > 95).

### qPSP is immune to noise in SWATH data

**Fig. 4:**
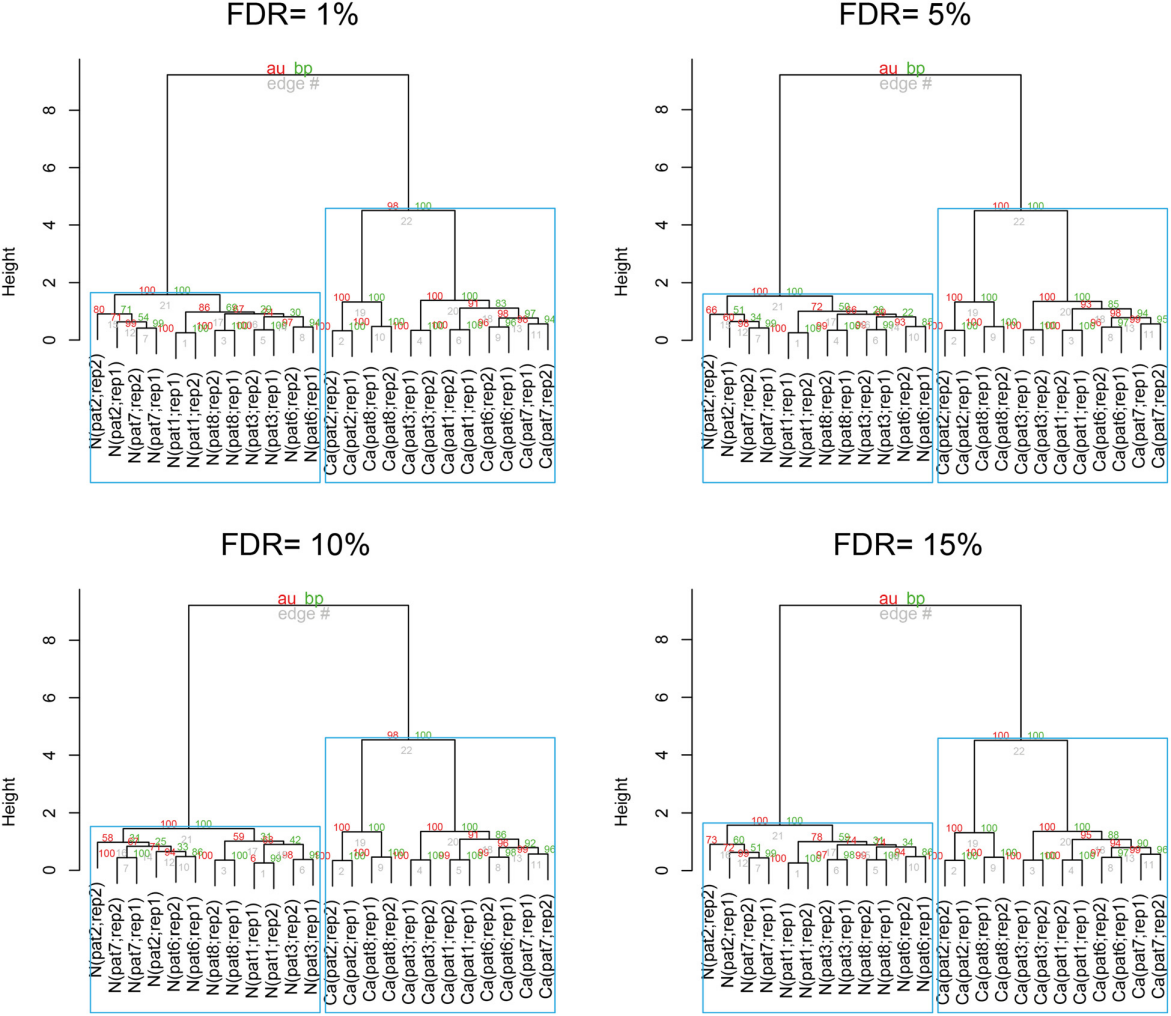
qPSP is stable at various FDR levels. This makes it useful for analyzing more proteins at the cost of introducing more noise into the system (1 % FDR, 180 clusters; 5 % FDR, 177; 10 % FDR, 178; 15 % FDR, 175)

To show that qPSP is immune to noise in real data, we adjusted the global false-discovery rates (FDR) in RC (since we have the raw spectra for RC but not CR) to 1, 5, 10 and 15 % levels, and repeated qPSP analysis on each of these FDR-adjusted RC datasets. Figure 4 showed HCL tree structures with significant groupings boxed in blue. The tree structure was maintained at all FDR levels although there were slight shifts in the significance levels of the AU scores. This indicated that qPSP was robust against increased noise levels in data. Therefore, qPSP could be deployed for improving sensitivity (recovering more low-confident but relevant proteins) while maintaining reasonable precision levels.

To understand the stability of the reported significant complexes, we cross-compared the set of reported significant complexes at 1, 5, 10 and 15 % FDR. It is expected that since the t-score is independent of the noise levels, the set of significant complexes should remain stable.

The set of significant complexes at each FDR level (*P* < = 0.05) are given in Additional file 4: Table S1. As expected, the significant complexes were stable and practically similar across different FDR levels. At higher noise levels, slightly fewer complexes were able to meet the significance threshold. Eight (out of 180) complexes varied in presence across all four FDR levels. All, with the exception of one, were found at the lesser significance level (0.025 < *P* < 0.05). Most of the variability was found at the 15 % FDR level. Of the remaining complexes, although there were mild fluctuations of the p-values across different FDR levels, there appeared to be no evidence of the p-values becoming less significant as the FDR levels increased (based on trends analysis of Additional file 4: Table S1).

Thus, in agreement with theoretical proof, qPSP is robust against noise.

### *qPSP* is more stable than standard network-enrichment analysis based on the hypergeometric test

**Fig. 5:**
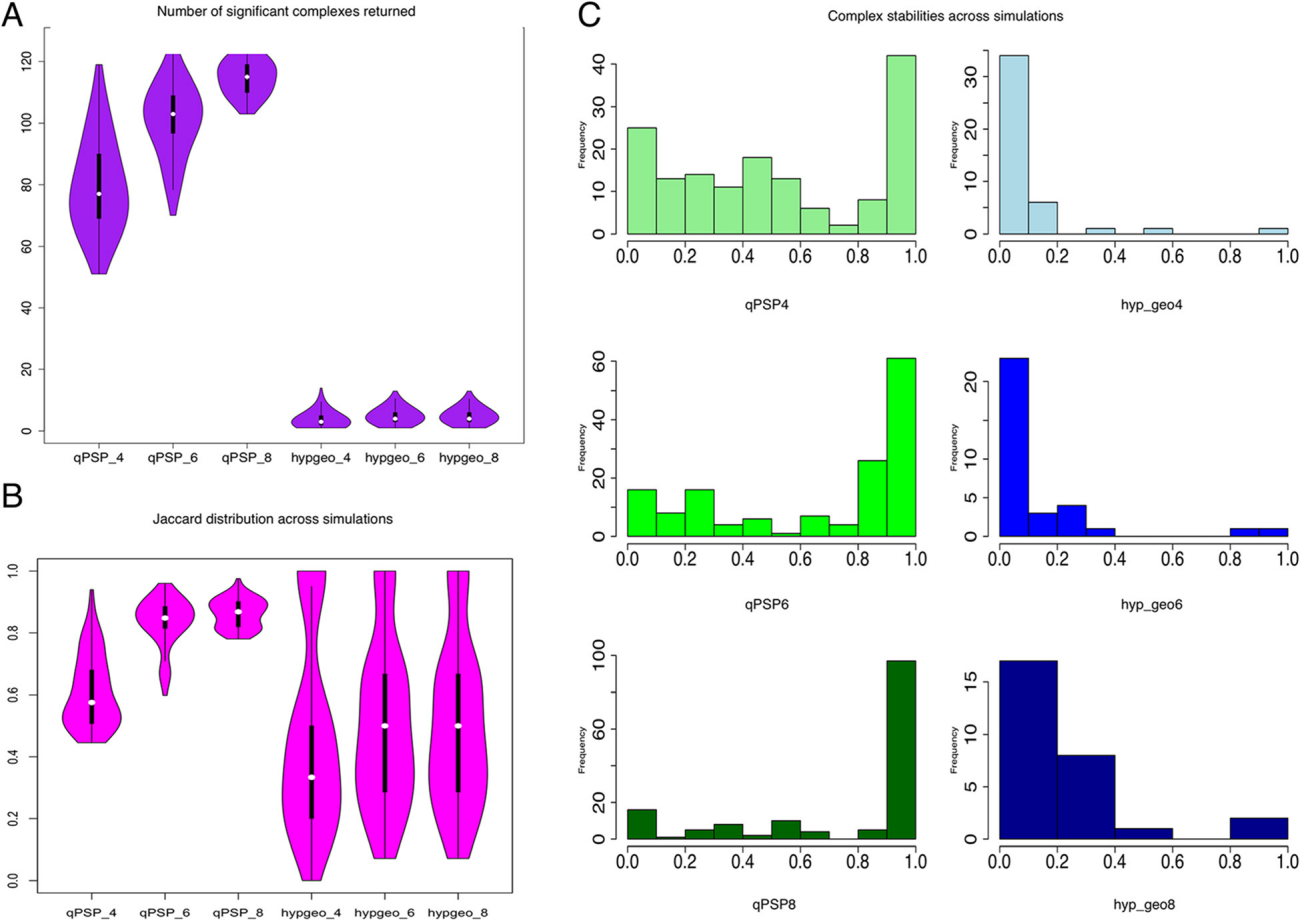
Stability analysis of qPSP and hyp_geo using bootstrap resampling in RC (Renal Cancer). **a** Distribution of number of significant complexes returned. Across various sampling sizes (4, 6 and 8), qPSP consistently reported more significant complexes than t-test selection for differential proteins followed by complex selection using the hypergeometric test (hyp_geo). **b** Simulation similarity comparisons. Pair-wise analysis of simulations to calculate the agreement levels (using Jaccard Score, 0 for complete disagreement, 1 for complete agreement) across complexes showed that qPSP was far more consistent than hyp_geo. **c** Complex persistency distribution. Distributions of significant complex agreements (On the x-axis, a score of 1 means complete persistence across all simulations, the y-axis is a frequency measurement, and its sum adds up to all complexes observed to be significant at least once). As sampling size increased, more complexes became more persistently represented across all simulations. However, this effect was much more pronounced for qPSP than hyp_geo. More importantly, the left skew for qPSP showed that most complexes were stable across samplings, while the right skew for hyp_geo showed that most of the significant complexes picked up were unstable

**Fig. 6:**
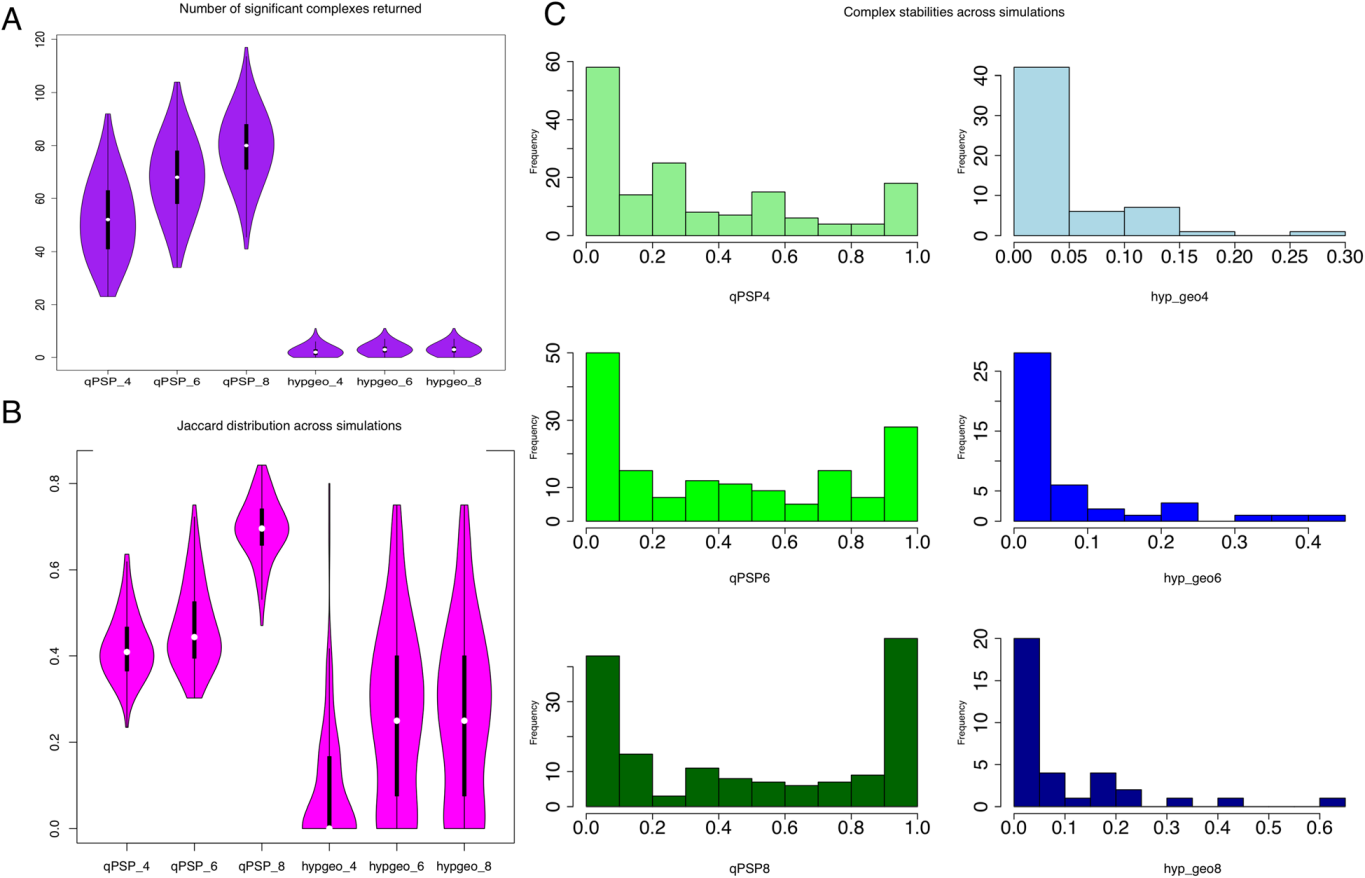
Stability analysis of qPSP and hyp_geo using bootstrap resampling in CR (Colorectal Cancer). **a** Distribution of number of significant complexes returned. Across various sampling sizes (4, 6 and 8), qPSP consistently reported more significant complexes than t-test selection for differential proteins followed by complex selection using the hypergeometric test (hyp_geo). The difference was sufficiently large to obviate the need for a statistical test. **b** Simulation similarity comparisons. Pair-wise analysis of simulations to calculate the agreement levels (using Jaccard Score, 0 for complete disagreement, 1 for complete agreement) across complexes showed that qPSP was far more consistent than hyp_geo. **c** Complex persistency distribution. Distributions of significant complex agreements (On the x-axis, a score of 1 means complete persistence across all simulations, the y-axis is a frequency measurement, and its sum adds up to all complexes observed to be significant at least once). As sampling size increased, more complexes became more persistently represented across all simulations. However, this effect was much more pronounced for qPSP than hyp_geo. More importantly, the left skew for qPSP showed that most complexes were stable across samplings, while the right skew for hyp_geo showed that most of the significant complexes picked up were unstable

For RC and CR, we compared qPSP to the hypergeometric enrichment method for subnet analysis. Figures 5a and 6a showed the distribution of significant complexes returned across 1000 simulations. At all sampling sizes, qPSP (4 to 8) returned significantly more complexes. This number increased as the sampling size increased. This was not unexpected, since the sample sizes involved were small. On the other hand, the significant complexes identified by hyp_geo (hyp_geo 4 to 8) were extremely low, averaging about 7 to 10 per simulation.

A mechanistic explanation for the observed increase of significant complexes by qPSP as sampling size increased is intuitive: Suppose the underlying differences between cancer and control are heterogeneous (let’s assume there are 5 causes). If we pick a small sample size, we would only observe a subset of the 5 causes. If only one sample is picked, then only one cause would be observed. The more samples that were picked, the more likely better coverage was obtained (to pick up more causes), and hence the complexes associated with these causes. The increase is expected to taper off once sufficient samples to observe all complexes associated with all underlying causes are obtained.

The stability of the significant complexes in qPSP and hyp_geo differed greatly as well. Figures 5b and 6b showed the distribution of agreement (Jaccard scores; 0 for complete disagreement and 1 for complete agreement) between simulations (pairwise). This perspective provided an indication of the level of agreement of the significant complexes between simulations. Although qPSP reported more significant complexes, these were stable relative to hyp_geo (Figs. 5b and 6b).

To examine complex instability from a complex-wise perspective, we summed each column of the binary matrix (each column is a complex, while each row is a simulation) and kept columns with sum greater than 0 (meaning that a simulation has reported this complex to be significant at least once), and divided the value by 1000 as a normalized value (Figs. 5c and 6c) such that a perfect value of 1 means that this complex is reported significant in all simulations while a value close to 0 means that the complex is unstably observed.

The frequency distribution of the column sums (in histogram form) provided a simple but effective visual representation for discerning whether the majority of significant complexes were stable or unstable. Figures 5c and 6c revealed that qPSP had a left skew---the majority of significant complexes were stable, and this stability improved as the sampling size increased. On the other hand, hyp_geo complexes had a right skew, showing that the majority of complexes were unstable. This distribution shape persisted even as sampling size increased showing no convergence. However, for CR, complex stability improved to a lesser extent as sampling size increased.

A valid concern is that qPSP is biased towards complexes that are enriched with high-abundance proteins. However, we argue that the benefit of having a reasonable number of consistent significant complexes is preferable to the downside of unstable and low number of significant complexes. Instability prevents generalizable interpretation of biological mechanism (since the significant proteins and complexes are unstable, they cannot be used for determining the true underlying biology), while low numbers of significant but true complexes provides too little information to make any kind of biological inference (the complexes may be correct, but there are not enough of them to establish a comprehensive analysis).

### Hypergeometric enrichment is a poorly reproducible method due to unstable t-test protein selection

Given how widely the hypergeometric test (hyp_geo) is used, its poor performance was surprising. Hence it is necessary to understand why it failed. Since RC appears to be less complex than CR, we investigate further using RC.

**Fig. 7:**
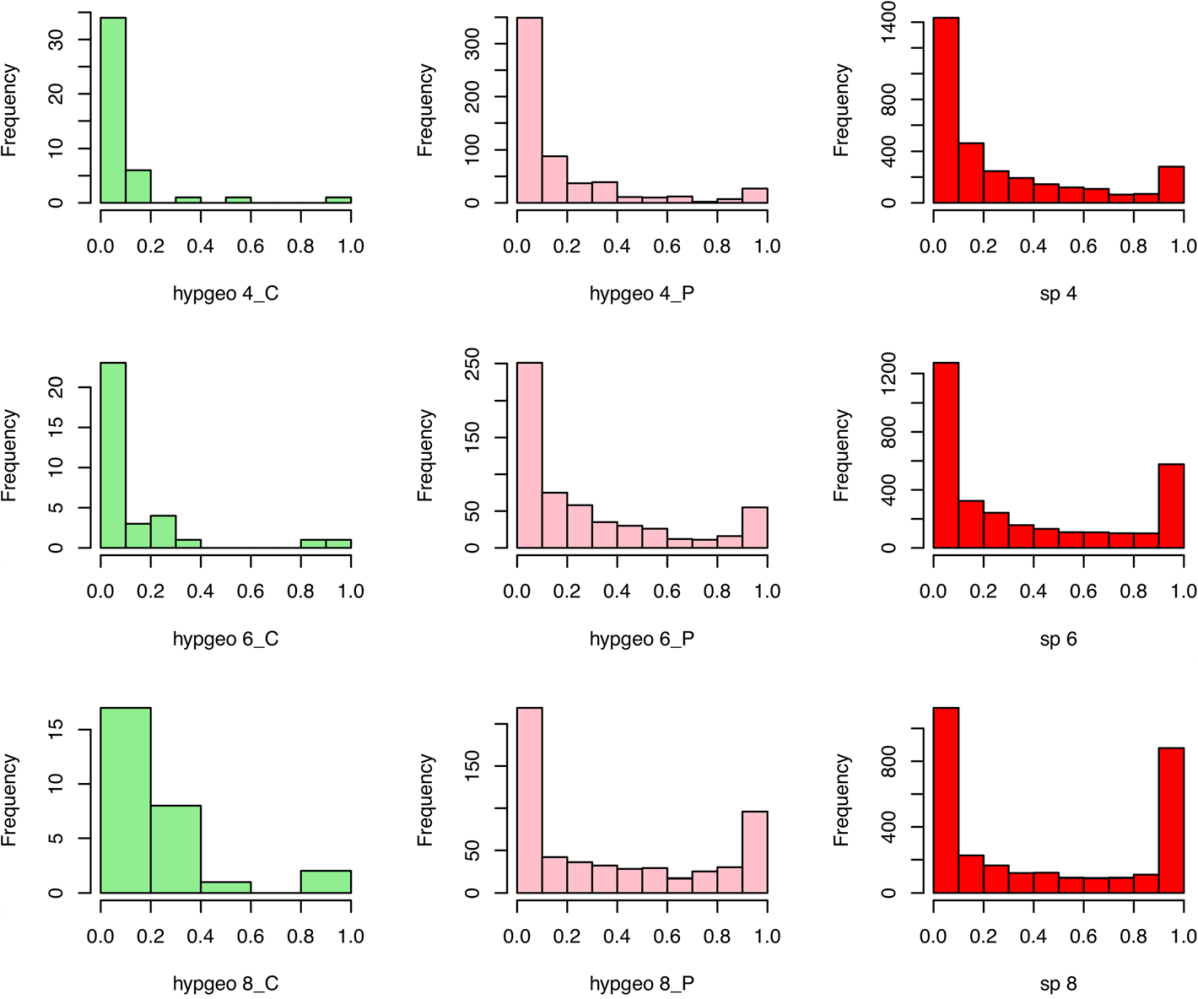
Hyp_geo’s complex selection stability mirrors closely to the t-test’s stability. hypgeo_n_C and hypgeo_n_P refers to the stability scores for the complexes and its constituent proteins respectively. Although increasing sampling size doesn’t improve the feature stability at the complex level (green), the feature stability from the corresponding proteins does improve and mirrors closely to the results from two-sample t-test protein selection (red)

To obtain a deeper understanding of feature-selection stability in hyp_geo, we plotted the stability histograms for complexes, their constituent proteins, and the stand-alone two-sample t-test (Fig. 7). In these histograms, a value near 0 on the x-axis means the feature is unstably observed while a value near 1 means the feature is observed across all random samplings. Separate histograms were generated for random samplings of sizes 4, 6 and 8.

It is interesting to note that the stability distribution of hyp_geo complexes mirrors closely that of their constituent proteins (i.e., those proteins that formed part of the complexes determined to be significant by the hypergeometric test itself). In turn, the stability distribution of these complex proteins resembles closely those proteins determined to be significant by the two-sample t-test. Given these resampling tests, there is no enrichment for highly stable t-test proteins within the hypergeometric-selected complexes. This means that the hypergeometric test is very sensitive to stability issues by the upstream t-test.

Since the hypergeometric test only makes a small number of statistically significant selections (approximately 20–30 complexes) compared to qPSP, it is of value to see if qPSP’s apparent feature-selection stability would be diminished if we restricted the selected features to the top 30 most significant instead of just those that met the p-value cutoff. Additional file 4: Figure S2 shows that even when restricting to the top 30 complexes in qPSP, the feature stability is still better than the hypergeometric test. This also implies that the p-values for each qPSP complex are relatively stable.

As a second fairness check, we restrict proteins to the top 20 % (in a manner similar to the top 20 % in qPSP) before running the hypergeometric test, Additional file 4: Figure S3 shows that restriction to the top proteins does not have strong effect on improving the performance of the hypergeometric enrichment method.

### qPSP uncovers informative protein complexes --- A case study in RC

In Fig. 3a, we were able to discriminate the severe and less-severe renal cancer phenotypes. Following this, we would like to achieve two more objectives: 1/briefly examine some of the complexes differentially expressed between severe and less severe cancer, and 2/show that the hit-rate measure in qPSP is an accurate summarization of the individual protein features across the complex.

**Fig. 8:**
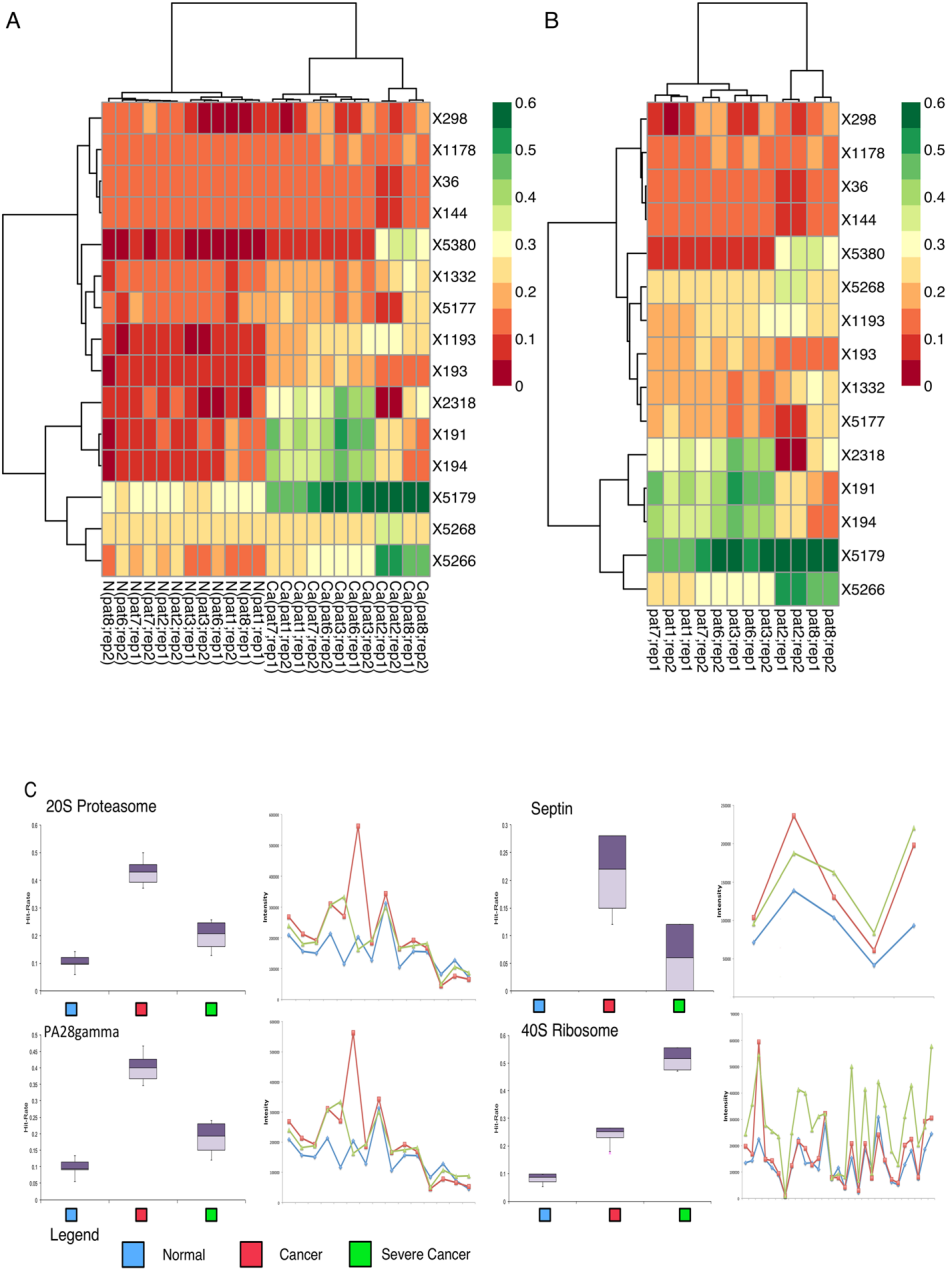
Comparative analysis of significant complexes between classes predicted by qPSP. **a** HCL (Euclidean Distance, Ward’s Linkage) clustering of normal and all cancer samples (columns) distributed over the PSP complex hit-rates (rows). **b** HCL clustering of 15 complexes across severe (in red box) and less-severe cancer phenotypes. Only a small subset of significant complexes was differential between severe and non-severe phenotypes. **c** Relationship between protein expressions and qPSP hit-rates. Hit-rates captured sensitively similar information as protein expression levels but is a more succinct representation

HCL (using Euclidean distance; Ward’s Linkage) between normal, and severe and less severe cancers showed the overall distribution of hit-rates for normal, severe and non-severe classes (Fig. 8a). We reduced the qPSP matrix to only include cancer samples and significant complexes (between normal and cancer), leaving 125 complexes. Next, we performed HCL clustering on this set, and removed all complexes that did not exhibit any difference between the two cancer phenotypes. This further reduced the complexes to 15.

The relationship between these complexes and the severe/less severe cancers are shown in Fig. 8b. We briefly examine 3 complexes out of the four shown in Fig. 8c---20S proteasome, Septin complex and 40S ribosome. Figure 8c further demonstrates that the hit-rates provide useful summarization that can distinguish between the normal, cancer and severe cancer samples. This measure is cleaner, and less complicated than looking at each discrete constituent gene expression.

There is some evidence in literature linking these complexes to renal cancer: For example, the 20S proteasome is a potential biomarker for non-metastasized breast cancer [38]. A recent study in 2012 demonstrated increased 20S proteasome levels in clear cell renal cancer, advanced disease, and poor prognosis [39]. The Septins have been shown to be fundamental for cytokinesis in many organisms and recognized as important components of the cytoskeleton [40]. Alterations in septin expression levels have been linked to various cancers, e.g. breast and leukemia [41, 42]. Interestingly, the 40S ribosome has higher hit rate in severe cases than less-severe cases. This complex is associated with metastasized RCC [43]. We related the hit-rates to the overall expression rate (Fig. 8c) and found that the results were consistent.

Details on the 15 differential complexes, which include their annotated functionalities, are provided in Table 1.

**Table 1:** RC Details on significant complexes differentiating normal and cancer phenotypes

CORUM ID	Complex name	Functionalities	qPSP pvalue
298	VEGF transcriptional complex	This complex regulates Src-dependent hypoxia-induced expression of VEGF in pancreatic and prostate carcinomas.	0.010
1178	BCOR complex (Ubiquitin E3 ligase)	Ubiquitin E3 ligases covalently attach ubiquitin to a lysine residue on a target protein. Polyubiquitination marks proteins for degradation by the proteasome. The BCOR complex contains E3 ligase activity for histone H2A. Monoubiquitylated H2A is present together with the BCOR complex at BCL6 target genes in B cells. This strongly suggests a role of BCOR as regulator of BCL6, a sequence-specific transcriptional repressor, that probably plays an important role in lymphomagenesis.	0.010
36	AP1 adaptor complex	This adaptor-related complex participates in a transport step different from that of AP-1. It may function at some trafficking step in the complex pathways between the TGN and the cell surface.	0.010
144	Gamma-BAR-AP1 complex	Protein targeting and endocytosis	0.012
5380	TRBP containing complex	Translation regulation	0.0016
5268	TNF-alpha/NF-kappa B signaling complex 7	Cytokine activity	0.02
1193	Rap1 complex	DNA repair; telomere maintenance	0
193	PA700-20S-PA28 complex	Proteasomal degradation	0
1332	Large Drosha complex	RNA processing	0.0001
5177	Polycystin-1 multiprotein complex	Kidney tissue differentiation	0.021
2318	ITGA6-ITGB4-Laminin10/12 complex	Cell adhesions	0.0001
191	20S proteasome	proteasomal degradation	0
194	PA28gamma-20S proteasome	proteasomal degradation	0
5179	NCOA6-DNA-PK-Ku-PARP1 complex	DNA repair	0
5266	TNF-alpha/NF-kappa B signaling complex 6	Cyokine activity	0

## Conclusions

qPSP is a powerful network contextualization approach for proteomics data. Using examples from DDA and DIA data, we demonstrated that qPSP produces strong yet stable class discrimination. It is robust against noise and makes reproducible feature (complex) selections, unlike subnet-enrichment analysis based on the hypergeometric test. Using the renal cancer dataset, we demonstrated that the qPSP hit-rate is a succinct summarization of contributing signals across multiple complex protein constituents. Given these qualities, qPSP is a powerful approach for analysis of clinical samples and selecting features for biomarker and drug target discovery.

## Reviewers’ comments

### Reviewer’s report 1

#### Reviewer name: Frank Eisenhaber, Bioinformatics Institute of Singapore

1. Formulation of the qPSP methodology

In its current form, qPSP basically utilizes the complex information (from the CORUM database) to form protein groups and then uses the protein abundance for the derivation of a new measure - hit rate per protein complex for any proteome sample. The hit rate measure is then used for subsequent statistic testing to elucidate enriched protein complexes between samples.

The current formulation did not consider the effect of protein complex stoichometry. To emphasize, high abundance of some proteins in a complex grouping does not necessarily imply that the same abundance of the complex. Hit rate/complex calculated from such cases can be grossly overestimated in complex abundance and skewed statistical significance. In contrast, the original PSP, which only uses presence or absence of the protein, has less bias in this regard. As a separate note, abundance of proteins is also independent of whether the actual protein interactions took place to form the complexes. Often, proteins are post-translationally modified so that the interactions do not take place though they are present in the cell. It is worrisome that the term “high abundance complexes” on the basis of protein abundance alone were being used in the text.

Taken together, the preceding assumptions made by qPSP are not trivial. At best, the hit rate is a predictive measure, but not a sufficient surrogate measure of protein complex formation and abundance. Its subsequent usage in statistical tests to identify enriched protein complexes creates the issue of nested predictions (predictions based on predictions); the proper usage of hypothesis testing is meant for testing differences in observed measures, not predictive measures.

2. Robustness of qPSP against noise: effects of t-score or pre-filtering?

From the theoretical argument, the authors presented a proof to show that t-score is independent of false-discovery rate (FDR = FP/TP + FP). Here, noise implicitly refers to the number of FP (false-positives). The proof seems incomplete since there was no discussion about effects of the pooled standard deviation term Su.v and the sample sizes n and m on the actual level of FP.

With respect to the two sample t-test, FP is equivalent to type I error (α). When the variances are unequal, the probability of type I error tends to be greater than the stated α. When n ≠ m, this probability will be greater than the stated α if the smaller sample is associated with the larger variance. In both cases, the level of FP (or noise) will be underestimated and the robustness of the t-test will be compromised as well. As such, qPSP does not seem impervious to FP arising from both unequal variances and sample sizes.

In actual applications, the robustness of qPSP to noise seems to stem from the pre-filtering step (i.e., the “alpha-stability analysis” that excludes protein hits below top 20 % of the ranking) rather than from the more elaborated hit rate/complex measure and FDR-independent t-statistics. There are two observations to note. First, the authors have shown that qPSP suffers in performance if the bottom hits were included in their RC-C study. Second, in the RC study, repeated qPSP analysis on various FDR-adjusted dataset has little impact on the classification tree results. The latter seems more likely a consequence of the pre-filtering step to create similarly truncated datasets (i.e., with similar level of FP hits removal) regardless of the initial FDR adjustments on the data.

3. Evaluation of the qPSP methodology

The evaluation of qPSP’s robustness reveals another issue. If the pre-filtering step alone in qPSP is sufficient to build similar clustering plots and hierarchical trees as shown throughout the article, then the inclusion of protein complex information and t-statistics adds little impact to the overall qPSP methodology.

Specifically to the RC dataset analysis, although the authors were able to show clear discrimination between the phenotypes in the HCL trees (as depicted in Fig. 3a), the qPSP methodology was unable to conclude any particular protein complex that strongly distinguishes between the severe versus less-severe phenotypes (as depicted by Fig. 8a). As such, the merits of including protein complex information to generate the hit rate for the t-statistics to improve biological interpretation have yet to be demonstrated.

Lastly, since qPSP is extended from PSP, the latter should be included in the performance comparison to demonstrate an improved performance.

Minor issues :

Section: qPSP is theoretically robust to experimental noise:

1. False-discovery rate is a more common terminology than false-detection rate.

2. It should be made clear that noise is referring to FP (false-positives) as a component of FDR (=FP/TP + FP).

3. It would be more readable to write the hit rate/complex computations as a formulation of vectors and matrix.

Section: implementation of qPSP:

Variable n has been used to twice. One instance is to describe a vector of complexes of length n, another use is as the size of samples n of phenotype B. The naming of the two variables should be distinct.

1/Arguments on not considering protein complex stoichiometry in qPSP

a) “not consider the effect of protein complex stoichometry…

We acknowledge and agree that this is a potential shortfall. However, whether the complex can or cannot be formed is something that cannot be directly addressed at the level of the proteomics screen. At the same time, we also agree that the simultaneous consideration of possible PTMs can be useful as additional information for refining the qPSP technique. In a typical proteomics screen however, increasing the screen for each PTMs (whether deterministic or stochastic), will greatly increase the library search space. Because there are a lot less expected matches relative to expected, it will drive up the false positive rates. This also means that we will have to increase the critical level accordingly but at the cost of further driving down proteome coverage. These analytical issues are interesting areas for further refinement and improvements but are upstream of qPSP.

For now, we are simply making the assumption that when the constituents of a complex are highly enriched/present in the proteomics screen, it is more likely that the complex can be formed. Currently, we are in fact looking into alternative assays that allow predictions of the strength of formation of various complexes. This can be potentially used as a weight for subsequent iterations of qPSP (we penalize those complexes that have low formation stability).

b) “hit rate is a predictive measure, but not a sufficient surrogate measure of protein complex formation and abundance”

The reviewers are correct to say that the hit-rate can be considered as a probabilistic indicator of protein complex formation and abundance. And in this regard, it is a form of predictive measure. But this is dependent on context and the manner in which these hit-rates are being used.

For now, we are just saying that each sample can be represented by a series of hit-rates against a feature vector of subnets. And that differential signal can be detected in this manner. We did not emphasize that the hit-rates themselves are predictive of the complex being formed or quantified. Moreover, in the last section of the manuscript, we considered the relation between hit-rates and the constituent protein quantitations. They correlated quite well in spite of the hit-rate being a summary measure.

c) “It is worrisome that the term “high abundance complexes” on the basis of protein abundance alone were being used in the text”.

We appreciate that the presence of “high abundance constituents” does not necessarily imply the presence of the “high abundance complex”. This term has only been used in one paragraph and was meant to make known a potential shortfall of the qPSP approach, namely, its tendency to pick complexes that is enriched for higher abundance proteins. We cannot observe all constituents in these complexes given that proteomics picks only about 3000 proteins in general, so for pithiness, we simply made the assumption that the complexes are high abundance. We will change this to the clearer term: “complexes enriched for high-abundance proteins” instead.

2/Robustness of qPSP against noise

a) “The proof seems incomplete since there was no discussion about effects of the pooled standard deviation term Su.v and the sample sizes n and m on the actual level of FP”.

The reviewer is correct that we made very specific assumptions wrt to the proof and it does not cover all potential scenarios as highlighted by the reviewer. Generally, we are saying that the actual level of FP comes from the false discovery rate for the proteomics data, which is estimated independently of S_u,v_ and m and n. Hence the unbiased s.d. S_u,v_ can be inferred based on k, m, n and the observed s.d. S_HA,HB_. Incidentally, the proof can be adapted to show the analogous result for t-score based on unequal variance.

We added some discussion on this issue in the main text of the manuscript. But briefly, in the unequal variance scenario,

$$ {S}_{HA,HB}=\frac{k}{1-r}\sqrt{\frac{{S_u}^2}{m}+\frac{{S_v}^2}{n}} $$

This can be plugged back into the t-expression:

$$ t\_ score=\frac{\overset{-}{HA}-\overset{-}{HB}}{\sqrt{\frac{{S_{HA}}^2}{m}+\frac{{S_{HB}}^2}{n}}}=\frac{E\left[u*k/\left(1-r\right)\right]-E\left[v*k/\left(1-r\right)\right]}{\left(\frac{k}{1-r}\right){S}_{u,v}\sqrt{\frac{1}{m}+\frac{1}{n}}} $$

And will still converge towards the same result as the proof in the original manuscript as k/1-r can still be factored out and cancelled.

b) “With respect to the two sample t-test, FP is equivalent to type I error (α). When the variances are unequal, the probability of type I error tends to be greater than the stated α. When n ≠ m, this probability will be greater than the stated α if the smaller sample is associated with the larger variance. In both cases, the level of FP (or noise) will be underestimated and the robustness of the t-test will be compromised as well. As such, qPSP does not seem impervious to FP arising from both unequal variances and sample sizes.”

This is indeed true. But maybe we can clarify this better. We are saying that qPSP is independent of r, which is the false discovery rate or uncertainty arising from the identification of proteins from proteomics. In typical proteomics, noise is typically very high, which normally requires imposing a very stringent false discovery rate (or false-detection rate) to restrict false matches (between 0.01 % to 1 % FDR). But this in turn, results in very low proteome coverage. Approaches that are able to circumvent this issue can be potentially very valuable.

The FP the reviewer is talking about here is regarding feature-selection based on the t-test, and not what we were discussing about above. The type I error was discussed in the earlier section “qPSP improves signal in proteomics data” where we used control data to test how many false positive complexes were predicted using resampling statistics. And yes, we agree, feature-selection instability where s.d. is very high and sample sizes very low is not something qPSP is immune against.

b) “the robustness of qPSP to noise seems to stem from the pre-filtering step (i.e., the “alpha-stability analysis” that excludes protein hits below top 20 % of the ranking) rather than from the more elaborated hit rate/complex measure and FDR-independent t-statistics. There are two observations to note. First, the authors have shown that qPSP suffers in performance if the bottom hits were included in their RC-C study. Second, in the RC study, repeated qPSP analysis on various FDR-adjusted dataset has little impact on the classification tree results. The latter seems more likely a consequence of the pre-filtering step to create similarly truncated datasets (i.e., with similar level of FP hits removal) regardless of the initial FDR adjustments on the data.”

qPSP is based on rank shifts amongst the extreme ranked proteins away from a defined alpha cutoff. As all samples have the same proteins reported, if we don’t use cutoffs, then there is no difference in the hit-rates. So the alpha cut-off is necessary.

We’ve shown that using the top 20 % of proteins seems to be a pretty good compromise. If alpha is set to too high e.g. top 1 %, then we have very few proteins to observe). If alpha is set too low, then we lose discriminatory power. Bottom alphas include low abundance proteins for which the rank shifts are more unstable, and less reliable. Their rank shifts are likely more due to chance than to phenotype-specific changes. In earlier work on genomics, we’ve also shown that in methods e.g. PFSNET and FSNET, using the top 20 % with complexes/subnets is an optimal alpha cutoff.

The FDR adjusted analyses reflect stability of analysis against low to high FDR levels given the same qPSP analysis procedure (alpha = top 20 %). This level was shown to be useful earlier in the paper, so we kept to it. We can definitely change the alpha levels but as we mentioned earlier, lowering the alpha will simply just increase the number of proteins, making sensitivity higher but also making it harder to find discriminatory signal amongst complexes. But this is not the aim of the section, which is to show that the results will hold constant in spite of changing the proteomics FDR levels.

3/Evaluation of the qPSP methodology

a) “ The evaluation of qPSP’s robustness reveals another issue. If the pre-filtering step alone in qPSP is sufficient to build similar clustering plots and hierarchical trees as shown throughout the article, then the inclusion of protein complex information and t-statistics adds little impact to the overall qPSP methodology.

Specifically to the RC dataset analysis, although the authors were able to show clear discrimination between the phenotypes in the HCL trees (as depicted in Fig. 3a), the qPSP methodology was unable to conclude any particular protein complex that strongly distinguishes between the severe versus less-severe phenotypes (as depicted by Fig. 8a). As such, the merits of including protein complex information to generate the hit rate for the t-statistics to improve biological interpretation have yet to be demonstrated.”

Prefiltering step in itself is not sufficient. In qPSP, the clustering is done based on the vector of hit-rates against the complexes, and never based on the protein expression levels themselves. Single protein expression based selection (e.g. t-test) is shown to be highly unstable and unreliable while the hypergeometric enrichment approach in itself also suffers from reproducibility issue. qPSP’s contextualization of protein expressions into complex hit-rates preserves the stability of feature-selection and reproducibility better than these two other commonly used approaches.

In Fig. 8, we simply showed all complexes without actually doing feature-selection. We have improved the presentation of the diagram such that differential complexes can be more easily shown. But the four examples in Fig. 8c show clearly that there are differences between normal, cancer, and severe cancer groups.

But we are limited by our biological knowledge (none of us are cancer biologists), and so we provide the list of differential complexes for real cancer experts to examine (Table 1). But proper confirmation in a specific biological problem, *in silico* and downstream biological validation is beyond the scope of this paper.

b) Lastly, since qPSP is extended from PSP, the latter should be included in the performance comparison to demonstrate an improved performance”.

PSP cannot work on data which has a complete matrix. This was one of the main reasons why we adapted qPSP using the fuzzified rank approach.

4/Terminologies

a) “False-discovery rate is a more common terminology than false-detection rate”.

We have made the changes.

b) “It should be made clear that noise is referring to FP (false-positives) as a component of FDR (=FP/TP + FP)”.

We have made the changes.

c) “It would be more readable to write the hit rate/complex computations as a formulation of vectors and matrix”.

We feel that this would have the effect of isolating non-computer science/non-math readers instead.

d) “Variable n has been used to twice. One instance is to describe a vector of complexes of length n, another use is as the size of samples n of phenotype B. The naming of the two variables should be distinct“.

We have made the changes.

### Reviewer’s report 2

This work is an extension of network contextualization methods for proteomic signature profiling by restricting the analysis to the top 20 % most abundant proteins and their complexes. Through reanalysis of 2 different cancer sets, the authors propose that the number of significantly detected complexes is higher than with classical hypergeometric network enrichment tests without increasing false positives and that the complex detections are robust to noise and small sample size.

The comparison does not seem very balanced given that all proteins (including low abundance ones that would increase noise) are considered for the hypergeometric tests (see major suggestion 1).

The network contextualization approach intuitively makes sense as by requiring multiple proteins from a protein complex to be identified before the complex and its proteins are considered present should give a more conservative picture of true protein identifications. This manuscript is dedicated to the addition of the criterion to only consider the top 20 % most abundant proteins (top 10 % weighted highest and staggered weights for the top 10-20 %) through which the conservatism is enhanced even further with some measurable advantages as mentioned above but the question is how useful is such a conservative view biologically when only restricting to most abundant protein complexes?

The first thing that comes to mind is that this approach will find and possibly be restricted to ribosomes and there-like which provides a rather limited chance of getting novel mechanistic insights. Unfortunately, many or most diseases’ causative mechanisms are expected to be more subtle and come from a variety of genes/proteins and pathways. Although the disease state could still correlate with ribosome abundance levels etc. it would likely be an indirect effect. This means that the ribosome levels may be used as biomarkers of disease states but much less so to provide a deeper understanding of disease mechanisms which could be targeted for drug interventions.

Indeed, 2 of 3 examples where they discuss biology of hits include ribosome complexes. However, the list of significant complexes in Additional file 4: Table S1 includes 180 complexes, some of which should be functionally much more specific and hence interesting. To help biologically knowledgeable readers judge better the biological significance and possibly get inspirations for further mechanistic studies, you may provide better and more info on the identified complexes (see major suggestion 2).

Major suggestion 1) Make a clean comparison of the different approaches to allow judging contributions of abundance criterion vs network contextualization. E.g. PSP vs. qPSP (same context method, different protein abundance criterion), qPSP vs hyp_geo_top20%abundant (different context method, same protein abundance criterion), qPSP vs hyp_geo_all (different context method, different protein abundance criterion). Parts of it are included already but should be presented more clearly.

Major suggestion 2) Show tables of at least the top 10 identified complexes unique to phenotype groups studied here in the main manuscript and longer lists in supplementary. Instead of complex identifier numbers use names (and list major protein constituents and basic known function). Also list, in a separate table, complexes uniquely identified by the qPSP approach compared to other methods tested here and discuss briefly any possibly interesting biological insights.

#### Reviewer name: Sebastian Maurer-Stroh, Bioinformatics Institute of Singapore

5/Major suggestion 1) Make a clean comparison of the different approaches to allow judging contributions of abundance criterion vs network contextualization. E.g. PSP vs. qPSP (same context method, different protein abundance criterion), qPSP vs hyp_geo_top20%abundant (different context method, same protein abundance criterion), qPSP vs hyp_geo_all (different context method, different protein abundance criterion). Parts of it are included already but should be presented more clearly.

PSP cannot be used here. It was originally meant for data with highly inconsistent protein identifications between samples and doesn’t work on data with a complete matrix.

For most papers, the default selection criteria for the hypergeometric test is to simply test proteins pre-selected by the t-test regardless of abundance. The suggestion to use hypergeometric test with the abundance criterion is an interesting one but not a setting that is typically used in application.

To satisfy the reviewer’s request, we have included this analysis under Additional file 4: Figure S3 and described it in the manuscript text.

6/Major suggestion 2) Show tables of at least the top 10 identified complexes unique to phenotype groups studied here in the main manuscript and longer lists in supplementary. Instead of complex identifier numbers use names (and list major protein constituents and basic known function). Also list, in a separate table, complexes uniquely identified by the qPSP approach compared to other methods tested here and discuss briefly any possibly interesting biological insights.

Thanks for the suggestion. There aren’t many interesting complexes so we just put them in Table 1 directly in the manuscript.

As for interesting features found by the other methods --- we found that they are hardly stable anyway. A comparative functional analysis of qPSP against the features these other methods selected seems to have very limited utility.

## Additional files

Additional file 1:
**Colorectal Cancer dataset (CR).** (TXT 4814 kb) Additional file 2:
**Renal replicates control dataset (RC-C).** (XLSX 1168 kb) Additional file 3:
**Renal cancer dataset (RC).** (TXT 485 kb) Additional file 4: Figure S1. RC-C HCL clustering using qPSP using various alpha thresholds (A) Using top and bottom ranked proteins (B) Using just top ranked proteins. Figure S2. qPSP restricted to top 30 complexes The histograms for CR and RC shows that even when restricted to the top 30 complexes, the p-values for each complex is fairly stable, and therefore, even across random samplings, are consistently observed. Figure S3. Stability analysis of qPSP and hyp_geo (top 20 %) using bootstrap resampling in RC (Renal Cancer). (A) Distribution of number of significant complexes returned. Across various sampling sizes (4, 6 and 8), qPSP consistently reported more significant complexes than t-test selection for differential proteins followed by complex selection using the hypergeometric test (hyp_geo). (B) Simulation similarity comparisons. Pair-wise analysis of simulations to calculate the agreement levels (using Jaccard Score, 0 for complete disagreement, 1 for complete agreement) across complexes showed that qPSP was far more consistent than hyp_geo. (C) Complex persistency distribution. Distributions of significant complex agreements (On the x-axis, a score of 1 means complete persistence across all simulations, the y-axis is a frequency measurement, and its sum adds up to all complexes observed to be significant at least once). **Table S1.** RC qPSP complexes and respective p-values changes by adjusted FDR levels. (DOCX 1144 kb)

## Abbreviations

CR: colorectal cancer
DIA: data-independent acquisition
DDA: data-dependent acquisition
FDR: false discovery rate
HCL: hierarchical clustering
hyp_geo: hypergeometric test
qPSP: quantitative proteomics signature profiling
RC: renal cancer
