# Supplementary material for: Quantitative proteomics signature profiling based on network contextualization
Source: Biol Direct. 2015 Dec 15;10:71. doi: 10.1186/s13062-015-0098-x (PMC4678536; doi:10.1186/s13062-015-0098-x)
Supplement: Additional file 4: Figure S1. — RC-C HCL clustering using qPSP using various alpha thresholds (A) Using top and bottom ranked proteins (B) Using just top ranked proteins. Figure S2. qPSP restricted to top 30 complexes The histograms for CR and RC shows that even when restricted to the top 30 complexes, the p-values for each complex is fairly stable, and therefore, even across random samplings, are consistently observed. Figure S3. Stability analysis of qPSP and hyp_geo (top 20 %) using bootstrap resampling in RC (Renal Cancer). (A) Distribution of number of significant complexes returned. Across various sampling sizes (4, 6 and 8), qPSP consistently reported more significant complexes than t-test selection for differential proteins followed by complex selection using the hypergeometric test (hyp_geo). (B) Simulation similarity comparisons. Pair-wise analysis of simulations to calculate the agreement levels (using Jaccard Score, 0 for complete disagreement, 1 for complete agreement) across complexes showed that qPSP was far more consistent than hyp_geo. (C) Complex persistency distribution. Distributions of significant complex agreements (On the x-axis, a score of 1 means complete persistence across all simulations, the y-axis is a frequency measurement, and its sum adds up to all complexes observed to be significant at least once). Table S1. RC qPSP complexes and respective p-values changes by adjusted FDR levels. (DOCX 1144 kb) [file 13062_2015_98_MOESM4_ESM.docx]

**Quantitative proteomics signature profiling based on network contextualization**

Wilson Wen Bin Goh^1,2,3,5*^, Tiannan Guo^3^, Ruedi Aebersold^3,4^, Limsoon Wong^5^

^1^ School of Pharmaceutical Science and Technology, Tianjin University, China

^2^ Center for Interdisciplinary Cardiovascular Sciences, Harvard Medical School, USA

^3^ Department of Biology, Institute of Molecular Systems Biology, ETH Zurich, Switzerland

^4^ Faculty of Science, University of Zurich, Switzerland

^5^ School of Computing, National University of Singapore, Singapore

*Corresponding Author: Wilson Wen Bin Goh, [wilson.goh@tju.edu.cn](mailto:wilson.goh@tju.edu.cn); [goh.informatics@gmail.com](mailto:goh.informatics@gmail.com)

Subject categories: Bioinformatics, Proteomics, Network Biology

Keywords: Proteomics, Networks, Quantitative Proteomics Signature Profiling (qPSP), Bioinformatics, SWATH, Systems Biology

**Supplementary Figures**

**Supplementary Figure 1** **RC-C HCL clustering using qPSP using various alpha thresholds** (A) Using top and bottom ranked proteins (B) Using just top ranked proteins

**Supplementary Figure 2** **qPSP restricted to top 30 complexes** The histograms for CR and RC shows that even when restricted to the top 30 complexes, the p-values for each complex is fairly stable, and therefore, even across random samplings, are consistently observed.

**Supplementary Figure 3** **Stability analysis of qPSP and hyp_geo (top 20%) using bootstrap resampling in RC (Renal Cancer).** (**A)** Distribution of number of significant complexes returned. Across various sampling sizes (4, 6 and 8), qPSP consistently reported more significant complexes than t-test selection for differential proteins followed by complex selection using the hypergeometric test (hyp_geo). (**B**) Simulation similarity comparisons. Pair-wise analysis of simulations to calculate the agreement levels (using Jaccard Score, 0 for complete disagreement, 1 for complete agreement) across complexes showed that qPSP was far more consistent than hyp_geo. (**C**) Complex persistency distribution. Distributions of significant complex agreements (On the x-axis, a score of 1 means complete persistence across all simulations, the y-axis is a frequency measurement, and its sum adds up to all complexes observed to be significant at least once).

**Supplementary Tables**

**Supplementary Table 1** **RC** **qPSP complexes and respective p-values changes by adjusted FDR levels**

| Complex | 1FDR | 5FDR | 10FDR | 15FDR |
| --- | --- | --- | --- | --- |
| 32 | 0.00010001 | 0.00020002 | 0.00030003 | 0.00040004 |
| 443 | 0.01250125 | 0.00910091 | 0.00920092 | 0.00970097 |
| 2174 | 0 | 0.00010001 | 0.00010001 | 0 |
| 5589 | 0.01120112 | 0.01310131 | 0.01070107 | 0.01230123 |
| 5611 | 0 | 0 | 0 | 0 |
| 3102 | 0.02630263 | 0.02860286 |  |  |
| 520 | 0.03070307 | 0.0240024 | 0.01640164 | 0.01530153 |
| 1004 | 0.00020002 | 0.00130013 | 0.00060006 | 0.00080008 |
| 1261 | 0.00280028 | 0.00170017 | 0.0010001 | 0.00080008 |
| 798 | 0.00130013 | 0.00170017 | 0.00170017 | 0.00170017 |
| 525 | 0.00130013 | 0.00170017 | 0.00170017 | 0.00170017 |
| 587 | 0.01820182 | 0.02020202 | 0.01840184 | 0.01930193 |
| 787 | 0.00130013 | 0.00170017 | 0.00170017 | 0.00170017 |
| 27 | 0 | 0 | 0 | 0 |
| 5233 | 0 | 0 | 0 | 0 |
| 194 | 0 | 0 | 0 | 0 |
| 298 | 0.00050005 | 0.00110011 | 0.00080008 | 0.00070007 |
| 5615 | 0 | 0 | 0 | 0 |
| 1186 | 0 | 0 | 0 | 0 |
| 714 | 0.01650165 | 0.02290229 | 0.02950295 | 0.03730373 |
| 822 | 0.02810281 | 0.03040304 | 0.02810281 | 0.02760276 |
| 888 | 0.01820182 | 0.02020202 | 0.01840184 | 0.01930193 |
| 1106 | 0.00010001 | 0.00090009 | 0.00050005 | 0.00060006 |
| TN10 | 0 | 0 | 0 | 0 |
| 5232 | 0 | 0 | 0 | 0 |
| 529 | 0.00130013 | 0.00170017 | 0.00170017 | 0.00170017 |
| 5386 | 0.04280428 | 0.03150315 | 0.0290029 | 0.03150315 |
| 846 | 0 | 0 | 0 | 0 |
| 753 | 0.00940094 | 0.00860086 |  |  |
| 2884 | 0 | 0 | 0 | 0 |
| 2721 | 0.00150015 | 0.00210021 | 0.00220022 | 0.00330033 |
| 2318 | 0.00110011 | 0.00080008 | 0.00080008 | 0.00190019 |
| 1239 | 0 | 0 | 0.00010001 | 0 |
| 208 | 0.01180118 | 0.01190119 | 0.01130113 | 0.01060106 |
| 726 | 0.00920092 | 0.01270127 | 0.01380138 | 0.01240124 |
| 5613 | 0 | 0 | 0 | 0 |
| 728 | 0.02940294 | 0.02660266 | 0.02520252 | 0.0250025 |
| 1150 | 0.01120112 | 0.01310131 | 0.01070107 | 0.01230123 |
| 3055 | 0 | 0.00010001 | 0 | 0.00010001 |
| 1181 | 0 | 0 | 0 | 0 |
| 889 | 0.01120112 | 0.01310131 | 0.01070107 | 0.01230123 |
| 3040 | 0 | 0 | 0 | 0 |
| 2203 | 0.0040004 | 0.00310031 | 0.00220022 | 0.00290029 |
| 649 | 0.01120112 | 0.01310131 | 0.01070107 | 0.01230123 |
| 211 | 0.01180118 | 0.01190119 | 0.01130113 | 0.01060106 |
| 5269 | 0.04110411 |  |  |  |
| 2851 | 0.01120112 | 0.01310131 | 0.01070107 | 0.01230123 |
| 5253 | 0.00090009 | 0.00070007 | 0.00040004 | 0.00060006 |
| 1191 | 0 | 0 | 0 | 0 |
| 1746 | 0 | 0 | 0 | 0 |
| 642 | 0.00360036 | 0.00460046 | 0.00330033 | 0.00310031 |
| 886 | 0.01120112 | 0.01310131 | 0.01070107 | 0.01230123 |
| 86 | 0 | 0 | 0.00010001 | 0 |
| 3066 | 0 | 0 | 0.00010001 | 0 |
| 247 | 0 | 0 | 0 | 0 |
| 1737 | 0.00880088 | 0.01130113 | 0.01110111 | 0.01040104 |
| 5231 | 0.00060006 | 0.00070007 | 0.00090009 | 0.00050005 |
| 5183 | 0 | 0 | 0.00010001 | 0.00010001 |
| 727 | 0.02940294 | 0.02660266 | 0.02520252 | 0.0250025 |
| 1111 | 0.00020002 | 0.00130013 | 0.00060006 | 0.00070007 |
| 320 | 0.01510151 | 0.01420142 | 0.01220122 | 0.01440144 |
| 1345 | 0 | 0 | 0 | 0 |
| 924 | 0 | 0 | 0 | 0 |
| 186 | 0.00710071 | 0.0050005 | 0.00490049 | 0.00440044 |
| 739 | 0 | 0 | 0 | 0 |
| 1231 | 0.00040004 | 0.00040004 | 0.00080008 | 0.00040004 |
| 732 | 0.01120112 | 0.01310131 | 0.01070107 | 0.01230123 |
| 811 | 0.00850085 | 0.00970097 | 0.01280128 | 0.01820182 |
| 650 | 0.01120112 | 0.01310131 | 0.01070107 | 0.01230123 |
| 3271 | 0.02370237 | 0.02820282 | 0.02440244 |  |
| 280 | 0.00020002 | 0 | 0 | 0 |
| 5211 | 0.04170417 | 0.04520452 | 0.04070407 | 0.04230423 |
| 2217 | 0 | 0 | 0 | 0 |
| 2599 | 0 | 0 | 0 | 0 |
| 112 | 0 | 0 | 0.00010001 | 0 |
| 191 | 0 | 0 | 0 | 0 |
| 4403 | 0.01240124 | 0.0080008 | 0.00680068 | 0.00530053 |
| 49 | 0 | 0 | 0 | 0 |
| 5196 | 0 | 0 | 0 | 0 |
| 834 | 0.00150015 | 0.00140014 | 0.0020002 | 0.00280028 |
| 646 | 0.01120112 | 0.01310131 | 0.01070107 | 0.01230123 |
| 181 | 0 | 0 | 0 | 0 |
| 5380 | 0.00080008 | 0.00090009 | 0.00090009 | 0.00130013 |
| 282 | 0.00920092 | 0.0210021 | 0.01950195 | 0.02150215 |
| 974 | 0.01120112 | 0.01310131 | 0.01070107 | 0.01230123 |
| 1237 | 0 | 0 | 0.00010001 | 0 |
| 5266 | 0.00160016 | 0.0020002 | 0.00130013 | 0.00190019 |
| 5286 | 0.03750375 |  |  |  |
| 1068 | 0.00130013 | 0.00150015 | 0.00090009 | 0.00130013 |
| 238 | 0 | 0 | 0.00010001 | 0 |
| 1149 | 0.01120112 | 0.01310131 | 0.01070107 | 0.01240124 |
| 1166 | 0.00130013 | 0.00170017 | 0.00170017 | 0.00170017 |
| 996 | 0.01120112 | 0.01310131 | 0.01070107 | 0.01230123 |
| 1252 | 0 | 0 | 0.00010001 | 0 |
| 486 | 0 | 0 | 0 | 0 |
| 561 | 0.0050005 | 0.00580058 | 0.00650065 | 0.00450045 |
| 5194 | 0 | 0 | 0 | 0 |
| 209 | 0.01180118 | 0.01190119 | 0.01130113 | 0.01060106 |
| 1227 | 0.00020002 | 0.00020002 | 0.00020002 | 0.00050005 |
| 3149 | 0.01120112 | 0.01310131 | 0.01070107 | 0.01230123 |
| 743 | 0.01120112 | 0.01310131 | 0.01070107 | 0.01230123 |
| 685 | 0.01820182 | 0.02020202 | 0.01840184 | 0.01930193 |
| 778 | 0 | 0 | 0 | 0 |
| 193 | 0 | 0 | 0 | 0 |
| 5604 | 0 | 0 | 0 | 0 |
| 324 | 0.04440444 | 0.04760476 |  |  |
| 1147 | 0 | 0 | 0 | 0 |
| 5596 | 0.01120112 | 0.01310131 | 0.01070107 | 0.01230123 |
| 1332 | 0 | 0 | 0 | 0 |
| 433 | 0 | 0 | 0 | 0 |
| 696 | 0.01120112 | 0.01310131 | 0.01070107 | 0.01230123 |
| 351 | 0 | 0 | 0 | 0 |
| 2939 | 0 | 0 | 0 | 0 |
| 713 | 0.00010001 | 0 | 0.00010001 | 0.00010001 |
| 2936 | 0 | 0 | 0 | 0 |
| 61 | 0.01820182 | 0.02020202 | 0.01840184 | 0.01930193 |
| 1413 | 0 | 0 | 0 | 0 |
| 430 | 0.00080008 | 0.00130013 | 0.00080008 | 0.00110011 |
| 835 | 0.00120012 | 0.00170017 | 0.00120012 | 0.00080008 |
| 2211 | 0 | 0 | 0 | 0 |
| 2319 | 0.00160016 | 0.00120012 | 0.00040004 | 0.0020002 |
| 5197 | 0.00060006 | 0.00070007 | 0.00090009 | 0.00050005 |
| 1183 | 0.00570057 | 0.00690069 | 0.00470047 | 0.00390039 |
| 1182 | 0.00520052 | 0.00340034 | 0.00350035 | 0.00180018 |
| 189 | 0.00040004 | 0.0010001 | 0.00070007 | 0.00090009 |
| 2757 | 0.00160016 | 0.00130013 | 0.00120012 | 0.00160016 |
| 5378 | 0.00150015 | 0.00190019 | 0.00180018 | 0.00120012 |
| 142 | 0.02370237 | 0.02820282 | 0.02440244 |  |
| 207 | 0.01180118 | 0.01190119 | 0.01130113 | 0.01060106 |
| 632 | 0.01820182 | 0.02020202 | 0.01840184 | 0.01930193 |
| 5209 | 0.00030003 | 0.00080008 | 0.00020002 | 0.00010001 |
| 5919 | 0 | 0 | 0 | 0 |
| 5199 | 0 | 0 | 0 | 0 |
| 725 | 0.00380038 | 0.00620062 | 0.00670067 | 0.00540054 |
| 149 | 0.00040004 | 0.0010001 | 0.00070007 | 0.00090009 |
| 304 | 0.00130013 | 0.00170017 | 0.00170017 | 0.00170017 |
| 563 | 0 | 0 | 0 | 0 |
| 476 | 0.02440244 | 0.02810281 | 0.02520252 | 0.0260026 |
| 210 | 0.01180118 | 0.01190119 | 0.01130113 | 0.01060106 |
| 2600 | 0.0150015 | 0.01340134 | 0.0090009 | 0.01040104 |
| 3082 | 0 | 0 | 0 | 0 |
| 306 | 0 | 0.00010001 | 0 | 0.00010001 |
| 470 | 0.02440244 | 0.02810281 | 0.02520252 | 0.0260026 |
| 1144 | 0.00810081 | 0.01060106 | 0.00930093 | 0.00860086 |
| 5400 | 0.0040004 | 0.00440044 | 0.00490049 | 0.00570057 |
| 116 | 0.00030003 | 0.00040004 | 0 | 0.00030003 |
| 5193 | 0 | 0 | 0 | 0 |
| 1097 | 0.00770077 | 0.00470047 | 0.00510051 | 0.00210021 |
| 338 | 0 | 0 | 0 | 0 |
| 1176 | 0.0090009 | 0.01090109 | 0.00870087 | 0.01010101 |
| 144 | 0 | 0.00010001 | 0 | 0 |
| 1155 | 0.00070007 | 0.00140014 | 0.00150015 | 0.00070007 |
| 570 | 0 | 0 | 0.00010001 | 0 |
| 300 | 0 | 0 | 0 | 0 |
| 1005 | 0.03870387 | 0.03450345 | 0.0390039 |  |
| 738 | 0.01120112 | 0.01310131 | 0.01070107 | 0.01230123 |
| 310 | 0 | 0 | 0 | 0 |
| 1230 | 0 | 0 | 0.00010001 | 0 |
| 192 | 0 | 0 | 0 | 0 |
| 614 | 0.01820182 | 0.02020202 | 0.01840184 | 0.01930193 |
| 5593 | 0.01120112 | 0.01310131 | 0.01070107 | 0.01230123 |
| 308 | 0.00030003 | 0.00050005 | 0.00040004 | 0.00070007 |
| 2727 | 0.01750175 | 0.01740174 | 0.01620162 | 0.01550155 |
| 5285 | 0.00030003 | 0.00080008 | 0.00050005 | 0.00070007 |
| 2829 | 0 | 0 | 0.00010001 | 0 |
| 3118 | 0.0010001 | 0.00120012 | 0.00080008 | 0.0010001 |
| 1226 | 0.00020002 | 0.00020002 | 0.00020002 | 0.00050005 |
| 1256 | 0.04280428 | 0.03150315 | 0.0290029 | 0.03150315 |
| 5609 | 0 | 0 | 0 | 0 |
| 626 | 0 | 0.00010001 | 0 | 0 |
| 5877 | 0.0020002 | 0.00190019 | 0.00160016 | 0.00210021 |
| 1223 | 0 | 0 | 0 | 0 |
| 1170 | 0.00130013 | 0.00170017 | 0.00170017 | 0.00170017 |
| 799 | 0.00130013 | 0.00170017 | 0.00170017 | 0.00170017 |
| 301 | 0 | 0 | 0 | 0 |
| 562 | 0.00030003 | 0.0010001 | 0.00090009 | 0.00110011 |
| 36 | 0 | 0.00010001 | 0 | 0 |
| 5230 | 0.00070007 | 0.00050005 | 0.00060006 | 0.00050005 |
| 5608 | 0 | 0.00020002 | 0.00020002 | 0 |
| 5268 | 0.04110411 |  |  |  |
| 781 | 0 | 0 | 0 | 0 |
| 1143 | 0.0010001 | 0.00120012 | 0.00080008 | 0.0010001 |

**Supplementary Data**

**Supplementary Data 1** **Colorectal Cancer dataset (CR)**

**Supplementary Data 2** **Renal replicates control dataset (RC-C)**

**Supplementary Data 3** **Renal cancer dataset (RC)**
